# Design of fractional evolutionary processing for reactive power planning with FACTS devices

**DOI:** 10.1038/s41598-020-79838-2

**Published:** 2021-01-12

**Authors:** Yasir Muhammad, Rizwan Akhtar, Rahimdad Khan, Farman Ullah, Muhammad Asif Zahoor Raja, J. A. Tenreiro Machado

**Affiliations:** 1 Department of Electrical and Computer Engineering, COMSATS University Islamabad, Wah Campus, Wah Cantt, 47040 Pakistan; 2 Department of Computer Science and Software Engineering, Pak-Austria Fachhochschule: Institute of Applied Science and Technology, Haripur, 22620 Pakistan; 3 Department of Electrical and Computer Engineering, COMSATS University Islamabad, Attock Campus, Attock, 43600 Pakistan; 4 Future Technology Research Center, National Yunlin University of Science and Technology, 123 University Road, Section 3, Douliou, Yunlin, 64002 Taiwan, ROC; 5 Department of Electrical Engineering, Polytechnic Institute of Porto, 4200-465 Porto, Portugal

**Keywords:** Energy science and technology, Engineering

## Abstract

Reactive power dispatch is a vital problem in the operation, planning and control of power system for obtaining a fixed economic load expedition. An optimal dispatch reduces the grid congestion through the minimization of the active power loss. This strategy involves adjusting the transformer tap settings, generator voltages and reactive power sources, such as flexible alternating current transmission systems (FACTS). The optimal dispatch improves the system security, voltage profile, power transfer capability and overall network efficiency. In the present work, a fractional evolutionary approach achieves the desired objectives of reactive power planning by incorporating FACTS devices. Two compensation arrangements are possible: the shunt type compensation, through Static Var compensator (SVC) and the series compensation through the Thyristor controlled series compensator (TCSC). The fractional order Darwinian Particle Swarm Optimization (FO-DPSO) is implemented on the standard IEEE 30, IEEE 57 and IEEE 118 bus test systems. The power flow analysis is used for determining the location of TCSC, while the voltage collapse proximity indication (VCPI) method identifies the location of the SVC. The superiority of the FO-DPSO is demonstrated by comparing the results with those obtained by other techniques in terms of measure of central tendency, variation indices and time complexity.

## Introduction

The optimal reactive power dispatch (ORPD) problem in coordination with flexible alternating current transmission system (FACTS) devices became a topic of growing research interest for the cost-effective operation and security of power systems^1–5^. The aim of the ORPD is to attain a fine tuning of the control variables for obtaining minimum transmission losses and acceptable voltage profiles while reducing the operational cost. Several voltage controlling devices are integrated in the power systems for voltage profile improvement, such as tap changing transformers^6^, shunt capacitors^7^, static VAR compensators (SVC), thyristor-controlled series compensators (TCSC) and thyristor-controlled phase shifters (TCPS)^8^. However, optimal allocation of the FACTS and setting their control variables poses a complex constraint optimization problem. Furthermore, such optimization has considerable influence on the effectiveness in system performance, by providing loss reduction, voltage profile improvement, network load ability enhancement, increased voltage stability and fuel cost reduction through optimal power flow^9–12^.

Due to these reasons we verify a growing interest to reach these objectives, namely with the incorporation of FACTS in legacy power systems. In power systems, weak buses were identified for the installation of FACTS through modal analysis. Moreover, the voltage collapse proximity indicator (VCPI) was tested for improving the voltage stability index. Optimal power flow (OPF) problems were solved by means of analytical methods^13^, genetic algorithms (GA)^14,15^, current injection^16^ and power injection model of FACTS^17^. Indeed, FACTS were installed at optimal locations to reduce voltage deviation^18^, active power loss^19^ and reactive power control^20^, or for security purposes such as in margin^21^ and congestion^22^ management. Several computational techniques were proposed for the optimal allocation of FACTS, namely fuzzy GA^23^, gravitational search algorithms^24^ and population-based techniques, such as differential evolution^25^, artificial bee colony with firefly^26^, quasi-oppositional chemical reaction optimization^27^, improved gravitational search algorithm^28^, ant lion optimizer^29^, whale optimization algorithm^30^, adaptive particle swarm optimization^31^ and chaotic krill herd algorithm^32^. These schemes have their own pros and cons and, therefore, it is important to explore the fractional swarming/evolutionary techniques, because these algorithms have not yet been exploited in the viewpoint of ORPD including FACTS.

In recent years, the implementation of fractional swarming and evolutionary computational strategies including fractional calculus in the internal structure of the optimizers was proposed. We can cite the PSO with fractional order velocity, fractional particle swarm optimization (FPSO), fractional order Darwinian PSO (FO-DPSO) and fractional order robotic PSO^33–38^. These methods were successfully applied in several problems including robot path controllers design^39^, image processing^40^, classification of hyperspectral images^41,42^, feature selection^43^, estimation of electromagnetic plane wave parameters^44^, adaptation of parameter for Kalman filtering algorithms^45^, localization and segmentation of optic disc^46^, design of discretized fractional order filters^47^ and land-cover monitoring^48^. Beside these application, we find also the design of PID controllers for AVR systems^49^, non-linear systems identification^50^, fractional robust control of coupled tank systems^51^, continuous nonlinear observer using sliding mode PID^52^ and design of power system stabilizer using GA-PSO^53^. These works point toward embedding the fractional calculus tools with the evolutionary strategies for optimization problems in the energy sector. This study explores the application of FO-DPSO for ORPD incorporating FACTS in electric power networks.

In various contingency situations, weak buses provide substantial evidences that they are responsible for voltage collapse. We start by applying power flow analysis and VCPI methods to detect the weak buses in the interconnected power system. Then, a new fractional version of the PSO, the FO-DPSO, is implemented as an efficient solver for ORPD problems. The algorithm involves the computation of control variables, including the values of SVC, TCSC, transformer tap positions and bus voltages, while satisfying the power demand. The voltage deviation, line loss minimization and system overall cost are considered as the objective functions, while observing the FO-DPSO execution. The highlights of the contribution can be summarized as:

- Novel application of the fractional swarming scheme for reliable solution of ORPD incorporating FACTS with optimization by means of the FO-DPSO.
- Application of the FO-DPSO on ORPD problems for reducing the overall cost, voltage deviation, and line loss minimization, while fulfilling of the load demand and operational constraints.
- Performance analysis of the FO-DPSO with different fractional orders conducted with ORPD problems.
- Statistical analysis, in terms of histograms, probability plots and learning curves, demonstrating the consistency, robustness, and stability of the proposed FO-DPSO.

The paper is structured as follows. “Objective functions of ORPD problem with FACTS devices” section formulates the fitness function for ORPD. “Mathematical model of FACTS” Section describes the mathematical modeling of FACTS and its influence in power system. “Weak bus detection for optimal positioning of FACTS” Section presents methods for the identification of weak buses. “Proposed methodology” Section gives an overview of the designed FO-DPSO, pseudocode and work flow diagram of the scheme. "Results and discussion" and "Statistical analysis" sections analyse several simulations with the proposed algorithm including a detailed comparison with other strategies and a statistical evaluation. Finally, the last “Conclusions” section summarizes the main conclusions.

## Objective functions of ORPD problem with FACTS devices

The locations of the SVC and the TCSC can be found by using the VCPI and the load flow analysis, respectively. Then the FO-DPSO is applied to optimize the control variables. This includes the reactive power generation, tap changer settings, and size of the TCSC and SVC considering the system evaluation functions. The expressions of the objective functions and constraints are given in the follow-up.

### Fitness function for power loss minimization

The fitness function for real power losses in power system is expressed as

1 $$\begin{aligned} \begin{array}{l} {\mathrm{Minimize}}\,\,{F_{PL}}({x_1},{x_2}) = {P_{Loss}}\\ \,\,\,\,\,\,\,\,\,\ = \sum \limits _{r = 1}^R {{g_r}\left[ {V_i^2 + V_j^2 - 2 \times {V_i} \times {V_j}\cos ({\delta _i} - {\delta _j})} \right] } \end{array} \end{aligned}$$

where $x_1$ and $x_2$ are defined as:

2 $$\begin{aligned} {x_2}= & {} \left[ \begin{array}{l} {T_1},{T_2},\ldots ,{T_N},\\ {V_{G1}},{V_{G2}},\ldots ,{V_{GN}},\\ {Q_{C1}},{Q_{C2}},\ldots ,{Q_{CN}},\\ SV{C_1},SV{C_2},\ldots ,SV{C_{N_{SVC}}},\\ TCS{C_1},TCS{C_2},\ldots ,TCS{C_{N_{TCSC}}} \end{array} \right] \end{aligned}$$

3 $$\begin{aligned} {x_1}= & {} \left[ \begin{array}{l} {Q_{G1}},{Q_{G2}},\ldots ,{Q_{GN}},\\ {V_{L1}},{V_{L2}},\ldots ,{V_{LN}},\\ {S_{L1}},{S_{L2}},\ldots ,{S_{LN}} \end{array} \right] \end{aligned}$$

In expressions (1) to (3) we have following variables and symbols:

- $F_1(x_1,x_2)$ consists of the loss minimization function.
- *R* represents the total number of transmission lines.
- $V_i$ and $V_j$ are the sending and receiving end voltages, respectively.
- $g_r$ stands for the line conductance.
- $\delta _i$ and $\delta _j$are the sending and receiving end voltage angles, respectively.
- $x_2$ denotes the vector of control variable consisting of transformers tap positions $\left( {{T_1},{T_2},\ldots ,{T_{{N_T}}}} \right)$, generators voltage magnitude $\left( {{V_{G1}},{V_{G2}},\ldots ,{V_{G{N_{PV}}}}} \right)$, reactive power compensators $\left( {{Q_{C1}},{Q_{C2}},\ldots ,{Q_{C{N_C}}}} \right)$, static VAR compensators $\left( {SV{C_1},SV{C_2},\ldots ,SV{C_{{N_{SVC}}}}} \right)$, thyristor controlled series capacitors $\left( {TCS{C_1},TCS{C_2},\ldots ,TCS{C_{{N_{TCSC}}}}} \right)$.
- $x_1$ denotes the vector of dependent variables that include the generator reactive power $\left( {{Q_{G1}},{Q_{G2}},\ldots ,{Q_{G{N_{PV}}}}} \right)$, load voltages $\left( {{V_{L1}},{V_{L2}},\ldots ,{V_{L{N_L}}}} \right)$ and line loading $\left( {{S_{L1}},{S_{L2}},\ldots ,{S_{L{N_L}}}} \right)$.

The allowable limits of the SVC and the TCSC are provided in Table 1. The equality constraints are defined as:

4 $$\begin{aligned}&{P_{Gi}} - {P_{Di}} - {V_i}\sum \limits _{j = 1}^{{N_{Bus}}} {{V_j}\left[ \begin{array}{l} {B_{ij}}\sin \left( {{\delta _i} - {\delta _j}} \right) \\ + {G_{ij}}\cos \left( {{\delta _i} - {\delta _j}} \right) \end{array} \right] } = 0 \end{aligned}$$

5 $$\begin{aligned}&{Q_{Gi}} - {Q_{Di}} - {V_i}\sum \limits _{j = 1}^{{N_{Bus}}} {{V_j}\left[ \begin{array}{l} {B_{ij}}\cos \left( {{\delta _i} - {\delta _j}} \right) \\ + {G_{ij}}\sin \left( {{\delta _i} - {\delta _j}} \right) \end{array} \right] } = 0 \end{aligned}$$

The inequality constraints consist of the transformer’s tap position settings, generators voltage and reactive power, and the SVC and TCSC boundaries as:

6 $$\begin{aligned}&T_i^{\min } \le {T_i} \le T_i^{\max },\,\,i = 1,2,\ldots ,{N_T} \end{aligned}$$

7 $$\begin{aligned}&Q_{Gi}^{\min } \le {Q_{Gi}} \le Q_{Gi}^{\max },\,\,i = 1,2,\ldots ,{N_{PV}} \end{aligned}$$

8 $$\begin{aligned}&V_{Gi}^{\min } \le {V_{Gi}} \le V_{Gi}^{\max },\,\,i = 1,2,\ldots ,{N_{PV}} \end{aligned}$$

9 $$\begin{aligned}&Q_{ci}^{\min } \le {Q_{ci}} \le Q_{ci}^{\max },i = 1,2,\ldots ,{N_c} \end{aligned}$$

10 $$\begin{aligned}&SVC_i^{\min } \le SV{C_i} \le SVC_i^{\max },i = 1,2,\ldots ,{N_{SVC}} \end{aligned}$$

11 $$\begin{aligned}&TCSC_i^{\min } \le TCS{C_i} \le TCSC_i^{\max },i = 1,2,\ldots ,{N_{TCSC}} \end{aligned}$$

here, $P_{G_i}$ and $P_{D_i}$ are the *i*th bus active power supply and demand, respectively, $Q_{G_i}$ and $Q_{D_i}$ represent the *i*th bus reactive power supply and demand, respectively, $N_T$, $N_{TCSC}$, $N_{SVC}$ and $N_c$ correspond to the number of transformers, TCSCs, SVCs and fixed shunt capacitors, respectively.

### Fitness function for voltage deviation ($V_D$)

Keeping a steady voltage profile in power system for secure operation is a challenging objective. Mathematically, the reduction of ($$V_D)$$ can be characterized as:

12 $$\begin{aligned} {V_D} = \sum \limits _{i = 1}^{{N_{Bus}}} {\left| {{V_i} - 1.0} \right| } \end{aligned}$$

here, $V_i$ is the voltage at *i*th and $N_{BUS}$ is number of buses.

### Fitness function for overall operating cost minimization

The fitness function for overall operating cost minimization combines two parts. The first part incorporates the investment of FACTS devices, whereas the second part represents the cost due to energy loss. Therefore, the aim is not only to reduce the cost of energy losses associated with the TCSC and SVC during minimization of power loss, but also to minimize the initial investment of these devices. Hence, the overall fitness function for cost minimization can be formulated as:

13 $$\begin{aligned} {C_{overall}} = {C_{FACTS}} + {C_{Energy}}, \end{aligned}$$

where

$$\begin{aligned} {C_{Energy}} = {P_{Loss}} \cdot 0.06 \cdot 1000 \cdot 365 \cdot 24 \end{aligned}$$

Hereafter, we fix the following values: cost due to energy loss 0.06 $/kWh, capital cost of shunt capacitor 1000$, hours in a day 24, days in a year 365. The provided cost data for $$C_{Energy}$$ is taken from reference^1,30^. The cost $$C_{FACTS}$$ of the FACTS devices is taken from the Siemens AG database^20^ and is specified as

14 $$\begin{aligned} {C_{FACTS}} = \alpha {s^2} + \beta s + \gamma \end{aligned}$$

where $\alpha$, $\beta$, and $\gamma$ are the cost coefficients and *S* is the operating range of the FACTS devices in MVAR. The limits and values can be seen in Tables 1 and 2.

**Table 1:** Boundaries of the control variables.

Control variables	IEEE 30 bus system		IEEE 57 bus system	
	Max	Min	Max	Min
SVC	0.20	0	0.20	0
TCSC	0.08	0	0.11	0
Transformers tap	1	0.9	1.05	0.9

**Table 2:** Cost coefficients TCSC and SVC.

FACTS devices	$\alpha$	$\beta$	$\gamma$
TCSC	0.0015	− 0.7130	153.75
SVC	0.0003	− 0.3051	127.38

## Mathematical model of FACTS

The solid-state devices provide an innovative concept in load flow control through network branch, fault reduction and reduced line losses, while keeping desired level of voltages^30^. This can be implemented by governing the network parameters including the current, voltage, phase angle, series and shunt impedances by incorporating FACTS in the electric power network. From the family of the FACTS, the TCSC and the SVC are used as stunt and series compensating devices, respectively. The mathematical model of TCSC and SVC along their influence after integrating into the network is discussed in the next sub-section.

### TCSC modelling

**Figure 1:**
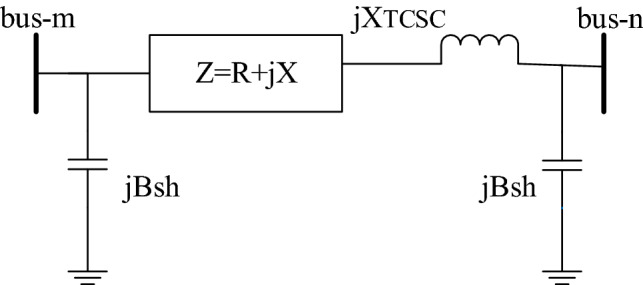
Static model of the TCSC.

The TCSC provides a variable reactive impedance equation $jX_c$ that can be altered above and below of the original impedance line. The power system static model equipped with TCSC between the *m*th to *n*th buses can be seen in Fig. 1. The power flow equations for the active and reactive components after coupling the TCSC are expressed, respectively, as^30^

15 $$\begin{aligned} {P_{mn}}= & {} + V_m^2{G_{mn}} - {V_m}{V_n}{G_{mn}}\cos ({\delta _m} - {\delta _n})\nonumber \\&- {V_m}{V_n}{B_{mn}}\sin ({\delta _m} - {\delta _n}) \end{aligned}$$

16 $$\begin{aligned} {Q_{mn}}= & {} - V_m^2{B_{mn}} - {V_m}{V_n}{G_{mn}}\sin ({\delta _m} - {\delta _n})\nonumber \\&+ {V_m}{V_n}{B_{mn}}\sin ({\delta _m} - {\delta _n}) \end{aligned}$$

likewise, the power (real and reactive) flow equations from the *n*th to *m*th buses can be formulated as

17 $$\begin{aligned} {P_{nm}}= & {} + V_n^2{G_{nm}} - {V_n}{V_m}{G_{nm}}\cos ({\delta _n} - {\delta _m})\nonumber \\&- {V_n}{V_m}{B_{nm}}\sin ({\delta _n} - {\delta _m}) \end{aligned}$$

18 $$\begin{aligned} {Q_{nm}}= & {} - V_n^2{B_{nm}} - {V_n}{V_m}{G_{nm}}\sin ({\delta _n} - {\delta _m})\nonumber \\&+ {V_n}{V_m}{B_{nm}}\sin ({\delta _n} - {\delta _m}) \end{aligned}$$

where the susceptance and conductance of the transmission line are given by ${B_{mn}} = \frac{{ - X - {X_{TCSC}}}}{{{R^2} + {{(X - {X_{TCSC}})}^2}}}$ and ${G_{mn}} = \frac{R}{{{R^2} + {{(X - {X_{TCSC}})}^2}}}$, respectively.

The modified $Y_{bus}$ equation matrix after the installation of the TCSC between the buses of the network is given by:

19 $$\begin{aligned} Y_{bus}^{TCSC} = {Y_{bus}} + \left[ {\begin{array}{*{20}{c}} 0&{}0&{}0&{} \cdots &{}0&{}0\\ 0&{}{\Delta {y_{sr}}}&{}0&{} \cdots &{}{ - \Delta {y_{sr}}}&{}0\\ 0&{}0&{}0&{} \cdots &{}0&{}0\\ \vdots &{} \vdots &{} \vdots &{} \cdots &{} \vdots &{}0\\ 0&{}{ - \Delta {y_{sr}}}&{}0&{} \cdots &{}{\Delta {y_{sr}}}&{}0\\ 0&{}0&{}0&{} \cdots &{}0&{}0 \end{array}} \right] \end{aligned}$$

Here, $\Delta {y_{sr}}$, is the change in admittance value after the installation of TCSC. These new entries of the line reactance affect the branch data due to the presence of TCSC.

### SVC modelling

**Figure 2:**
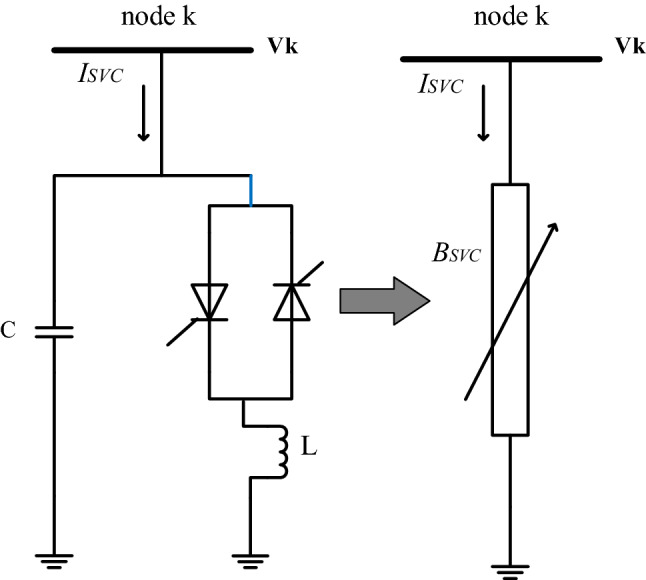
Static SVC model.

The SVC can inject and absorb reactive power to and from the bus bar by coupling different topologies of inductors and capacitors in shunt. The reactive power flow is governed by the phase-controlled operation of thyristor valve to quickly remove or add parallel connected capacitor and reactors. The equivalent model of the SVC that can also be implemented as a parallel integrated variable susceptance $B_{SVC}$ at any given bus-k is depicted in Fig. 2. The reactive power flow from the SVC into the bus can be written as^30^:

20 $$\begin{aligned} {Q_{svc}} = {B_{SVC}}{V^2} \end{aligned}$$

where *V* is the amplitude of bus voltage where the compensator is installed. The modified admittance ($Y_{bus}$) matrix after installation of the SVC at a given bus is expressed as:

21 $$\begin{aligned} Y_{bus}^{SVC} = {Y_{bus}} + \left[ {\begin{array}{*{20}{c}} 0&{}\quad 0&{}\quad 0&{}\quad \cdots &{}\quad 0&{}\quad 0\\ 0&{}\quad {{Y_{shunt}}}&{}\quad 0&{}\quad \cdots &{}\quad 0&{}\quad 0\\ 0&{}\quad 0&{}\quad 0&{}\quad \cdots &{}\quad 0&{}\quad 0\\ \vdots &{}\quad \vdots &{}\quad \vdots &{}\quad \cdots &{}\quad \vdots &{}\quad 0\\ 0&{}\quad 0&{}\quad 0&{} \quad \cdots &{}\quad 0&{}\quad 0\\ 0&{}\quad 0&{}\quad 0&{}\quad \cdots &{}\quad 0&{}\quad 0 \end{array}} \right] \end{aligned}$$

Here, $\Delta {Y_{shunt}}$, is the shunt admittance of SVC. These modified values in the admittance matrix due to the SVC affects the bus data.

## Weak bus detection for optimal positioning of FACTS

The main aim of a weak bus recognition is to obtain the best possible position of the FACTS devices for providing proper reactive power provision at the suitable locations. This action affects the natural characteristics of the electrical transmission lines, provides better voltage profile, increases the power transfer capacity, reduces line losses and solves problems related to voltage instability^31^. The method used in the present work for weak bus identification involves the load flow analysis through single line diagram.

### Voltage collapse proximity indication (VCPI)

The maximum power transfer theorem provides the basis of the VCPI technique^30^ for a line. Consider a constant voltage source $V_{s}$ with internal impedance $Z_s \angle \theta$ that is feeding a load with impedance $Z_L \angle \varphi$. According to this theorem, the maximum power flow occurs when the ratio of impedances $Z_L/Z_s$ equals 1. This result is used as voltage collapse predictor.

In order to simplify the problem and to maintain the accuracy level it is useful to keep $\varphi$ constant while considering a variable load impedance. For each increase of the load demand, it results that the current increases and $Z_L$ decreases. These combined effects further increase the line drop and decrease the voltage at the receiving end as follows:

22 $$\begin{aligned} I= & {} \frac{{{V_s}}}{{\sqrt{[{{({Z_s}\sin \theta + {Z_L}\sin \varphi )}^2} + {{({Z_s}\cos \theta + {Z_L}\cos \varphi )}^2}]} }} \end{aligned}$$

23 $$\begin{aligned} {V_r}= & {} {Z_L}I \end{aligned}$$

For

24 $$\begin{aligned} {V_r} = \frac{{{Z_r}}}{{{Z_s}}}\frac{{{V_s}}}{{\sqrt{1 + {{\left( {\frac{{{Z_r}}}{{{Z_s}}}} \right) }^2} + 2\left( {\frac{{{Z_r}}}{{{Z_s}}}} \right) \cos \left( {\theta - \varphi } \right) } }} \end{aligned}$$

the line power loss is given by

25 $$\begin{aligned} {P_l} = \frac{{V_{s}^{2}}\big /{Z_s}}{{\sqrt{1 + {{\left( {\frac{{{Z_r}}}{{{Z_s}}}} \right) }^2} + 2\left( {\frac{{{Z_r}}}{{{Z_s}}}} \right) \cos \left( {\theta - \varphi } \right) } }}\cos \varphi \end{aligned}$$

and the receiving end power by

26 $$\begin{aligned} {P_r}= & {} {V_r}I\cos \phi \end{aligned}$$

27 $$\begin{aligned} {P_r}= & {} \frac{V_s^2 \big / Z_s}{{\sqrt{1 + {{\left( {\frac{{{Z_r}}}{{{Z_s}}}} \right) }^2} + 2\left( {\frac{{{Z_r}}}{{{Z_s}}}} \right) \cos \left( {\theta - \varphi } \right) } }}\frac{{{Z_r}}}{{{Z_s}}}\cos \varphi \end{aligned}$$

The maximum power ${P_r}$ can be attained by applying the boundary conditions $(\partial {P_r}/\partial {Z_L}) = 0$, which implies that ${Z_L}/{Z_s} = 1$. By replacing this in Eq. (27), the maximum power transfer capacity results

28 $$\begin{aligned} {P_r} = \frac{{\cos \varphi }}{{4{{\cos }^2}\left( {\frac{{\theta - \varphi }}{2}} \right) }}\frac{{V_s^2}}{{{Z_s}}}. \end{aligned}$$

the line maximum power transfer forms the conceptual basis of VCPI and, therefore, it can be written as

29 $$\begin{aligned} VCPI = \frac{{{P_r}}}{{{P_{r(\max )}}}}, \end{aligned}$$

with a value that should be lower than one for a stable system. When this value is approaching the unity, for any bus, it means that it is getting closer to instability. This bus is identified as weak bus and designated as the best possible location for the SVC installation.

### Load flow analysis

The reactive power flow can be computed and those lines that transfer the higher value can be identified. The buses where the branch ends are referred as weak buses and the TCSC are installed between such buses.

The procedural steps for finding the TCSC locations are as follow:

1. Load bus and line of the test system.
2. Generate the Y-bus matrix.
3. Compute the angle and voltage of each bus using the Newton Raphson technique.
4. Compute the reactive power (Q) and active power (P) in each line using load-flow technique.
5. Pick the line/branch with maximum Q.
6. Conditional check: If the designated branch is a slack bus (reference bus), or if it is linked to a generator (generator bus), then repeat step 5, else switch to step 7.
7. The branch end-point or bus is designated for the position of the TCSC.

## Proposed methodology

**Figure 3:**
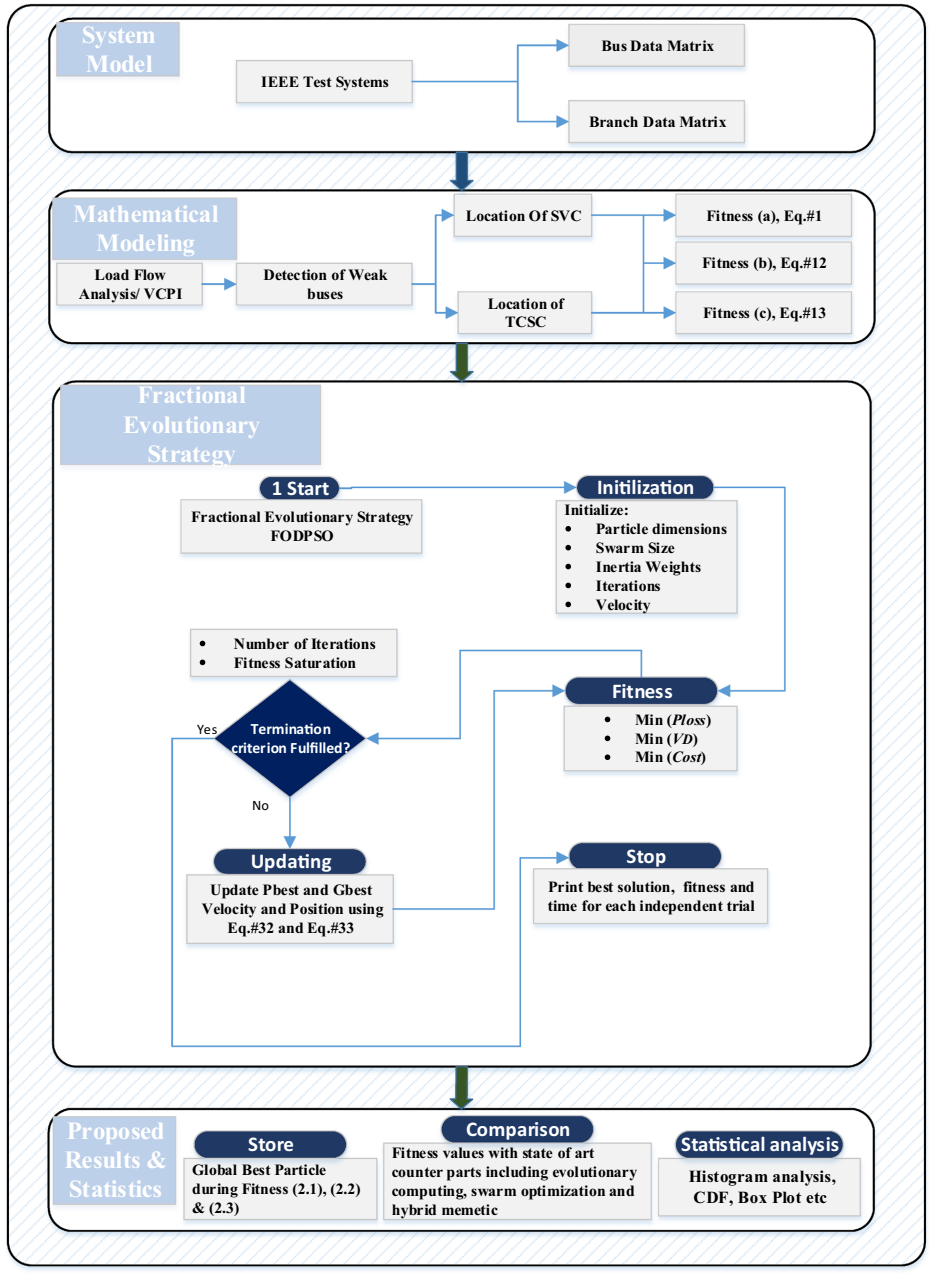
Overall flow diagram of the FO-DPSO for ORPD incorporating FACTS.

The proposed methodology includes two phases. In the first, an introductory overview of the FO-DPSO is presented. In the second phase the computational strategy in terms of the processing block structure and pseudocode are provided for ORPD incorporating FACTS. The overall workflow schematic of the presented technique is depicted in Fig. 3. The idea is to develop a technique based on the FO-DPSO for optimal sizing of the SVC and TCSC with appropriate placement in the inter connected power system, while minimizing the overall operational cost, power loss and voltage deviation of the IEEE standard test systems.

### Introduction of the FO-DPSO

The Darwinian PSO (DPSO) is an evolutionary mechanism that improves the standard PSO by increasing its capability to escape from local optimum either by natural assortment, or by persisting those with high fitness values. The performance of DPSO is superior to the one exhibited by the PSO, but has the disadvantage of a higher computational complexity. Pires et al. combined the DPSO with the concept of fractional calculus (FC) to improve learning ability of the DPSO mechanism by designing the Fractional Order Darwinian PSO (FO-DPSO)^54^.

The development of FC and its application in engineering problems has proved its importance due to heredity and long memory effects in many phenomena and systems^36^. The integro-differential operator defined by the Grünwald-Letnikov, Caputo and Riemann–Liouville formulations are classical expressions that are adopted in science and engineering. The Grüwald-Letnikov interpretation of the fractional derivative can be expressed as^35^

30 $$\begin{aligned} {D^\alpha }[f(t)] = \mathop {\lim }\limits _{h \rightarrow 0} \left[ {\frac{1}{{{h^\alpha }}}\sum \limits _{m = 0}^\infty {\frac{{{{\left( { - 1} \right) }^k}\Gamma \left( {\alpha + 1} \right) f\left( {t - kh} \right) }}{{\Gamma \left( {k + 1} \right) \Gamma \left( {\alpha - k + 1} \right) }}} } \right] , \end{aligned}$$

hereafter, *h* is sampling interval, $\alpha$ denotes the fractional order and $\Gamma$ stands for the Euler gamma function. In the present study we adopt the discrete time approximation

31 $$\begin{aligned} {D^\alpha }[f(t)] = \frac{1}{{{T^\alpha }}}\sum \limits _{m = 0}^r {\frac{{{{\left( { - 1} \right) }^k}\Gamma \left( {\alpha + 1} \right) f\left( {t - kT} \right) }}{{\Gamma \left( {k + 1} \right) \Gamma \left( {\alpha - k + 1} \right) }}}, \end{aligned}$$

where *T* corresponds to the sampling period and *r* is the truncation order. Considering $r = 4$, that is, adopting the first four terms for the expression (5.4.2) of differential derivatives, the velocity update equation for FO-DPSO is transformed from that of conventional PSO and is given for *n*th particle as:

32 $$\begin{aligned} v_{t + 1}^n= & {} \alpha v_t^n + \frac{1}{2}\alpha (1 - \alpha )v_{t - 1}^n + \frac{1}{6}\alpha (1 - \alpha )(2 - \alpha )v_{t - 2}^n\nonumber \\&+ \frac{1}{{24}}\alpha (1 - \alpha )(2 - \alpha )(3 - \alpha )v_{t - 3}^n\nonumber \\&+ {\phi _1}{r_1}(LB_t^n - s_t^n) + {\phi _2}{r_2}(GB_t^n - s_t^n) \end{aligned}$$

and position update is given as:

33 $$\begin{aligned} x_{t + 1}^n = x_t^n + v_{t + 1}^n, \end{aligned}$$

where *x* is the position vector of *n*th particle with velocity *v*, *t* is the flight index, and $\Phi _1$ and $\Phi _2$ are the personal best and global best acceleration constant, respectively. Moreover, *S* represents the swarm consisting of *m* particles, i.e., $x_1, x_2, x_m$, $r_1$ and $r_2$ are random numbers between 0 and 1, and *G* and *L* are the global and local best position vector in the swarm, respectively.

Equation (32) shows that the canonical PSO is a particular scenarios of the FO-DPSO with order of derivative $\alpha = 1$, i.e., without “memory”. In the literature, there is no specific method to find out the best fractional orders $\alpha$. The searching of the appropriate fractional order $\alpha$ for optimal performance of the fractional evolutionary/swarming techniques for a specific objective function is usually conducted by means of a stochastic procedure, i.e., the best performance of order $\alpha$ based on the statistics. The interpretation of the fractional order $\alpha$ used in the optimization using the fractional PSO and a possible justification through physics is always a complex task. The traditional practice is to adopt the Monte-Carlo simulations-based statistics to select the order $\alpha$ that perform best on a problem-oriented specific fitness function.

In addition, the results depend on the fractional order $\alpha$ depending on the problem and function convergence rate varies for different $\alpha$ and each scenario. In^55^, the convergence rate with respect to $\alpha$ was studied. In this case, the best results were obtained at lower orders for different test functions. In^35^, a faster convergence rate was attained at $\alpha$ in the range [0.5, 0.8]. In spite of these difficulties, all studies endorsed that adopting a fractional order provides better results in comparison with the integer case. In addition, each optimization scenario may have a different optimal value of $\alpha$. Afterwards, the FO-DPSO is adopted as a significant fractional evolutionary strategy considering the best $\alpha$, that is evaluated and selected using Monte-Carlo simulations-based statistics for each objective function.

### Application of FO-DPSO for ORPD incorporating FACTS

The key modification adopted in the FO-DPSO is the velocity update in the standard PSO since the fractional derivative is included in the algorithm. The global optimization effectiveness of FO-DPSO is explored for finding the best size of the TCSC, SVC, tap values and generators reactive power output in the IEEE-30, IEEE-57 and IEEE-118 buses power systems, while reducing the line losses, voltage deviation and overall cost.

The steps for the evaluation of control variables using FO-DPSO are given in Algorithm 1, and the parameter settings of FO-DPSO are documented in Table 3.

**Table 3:** Parameter settings of the FO-DPSO algorithm.

Parameters	IEEE 30 Bus (19 variables)	IEEE 57 Bus (25 variables)	IEEE 118 Bus (80 variables)
Particle dimensions or variables	19	25	80
Swarm, set of particles	50	50	50
Fractional order	0.6	0.6	0.3
Inertia weight	0.9–0.2	0.9–0.2	0.9–0.2
Global acceleration factor	0.1–0.9	0.1–0.9	0.1–0.9
Local acceleration factor	0.9–0.1	0.9–0.1	0.9–0.1
Iterations or cycles for statistics	80	180	50
$V_{max}$	2	2	2


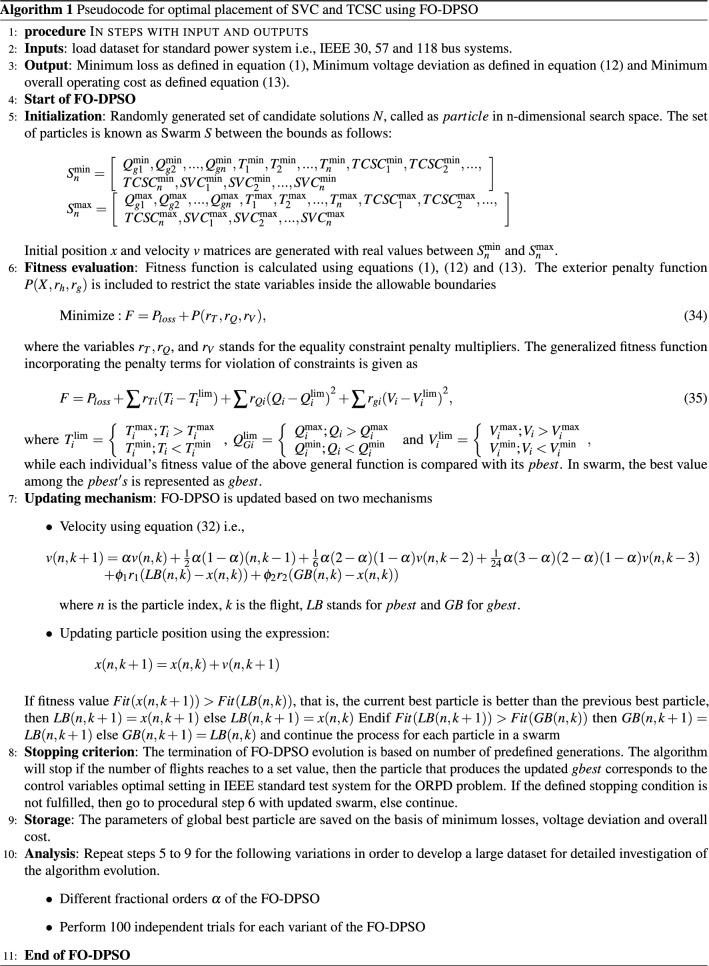


## Results and discussion

The validity and applicability of the FO-DPSO is analyzed for the reactive power scheduling in the IEEE 30, IEEE 57 and IEEE 118 buses while incorporating the SVC and TCSC devices at weak buses. We tested the performance of designed fractional order DPSO technique on a consistent similar strategy as reported in recent articles^35,55^ where the fractional order is taken between 0 and 1 (i.e., [0.1, 0.2,..., 1.0]). The theoretical and simulation analyses are presented for 10 values of the fractional order including the integer order case, i.e., $\alpha$ = 1, where the fractional DPSO transformed to standard DPSO. The search for the fractional order for optimal performance of fractional evolutionary/swarming technique is normally conducted on stochastic procedure, namely on the basis of statistics. Indeed the selection of $\alpha$ with a clear justification through physics is always difficult and Monte-Carlo statistics are used to select the order which performs best. To evaluate the optimization robustness of the FO-DPSO, a statistical analysis is performed for 100 trials during all test cases.

### Test case 1

**Figure 4:**
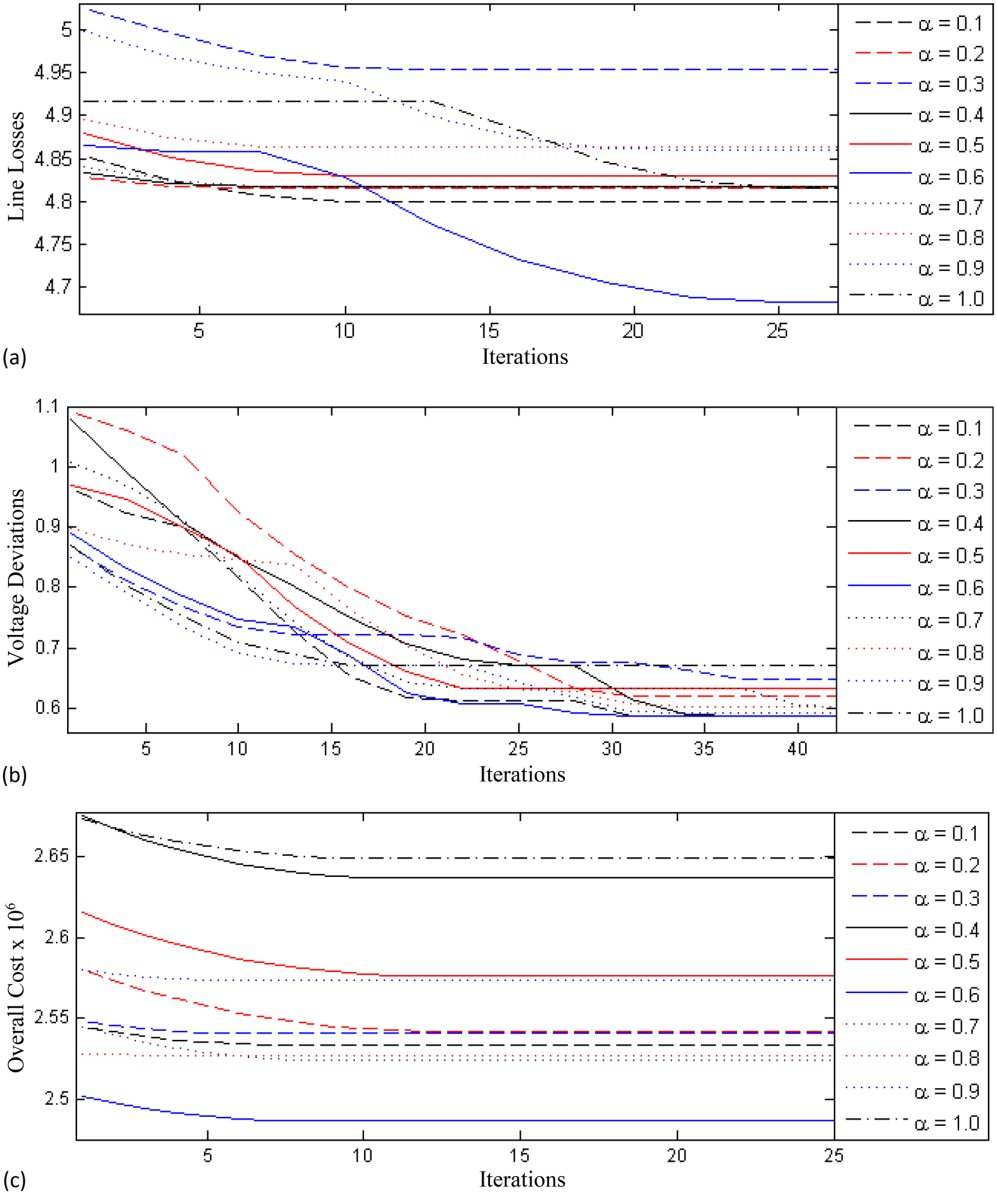
Learning curves for test case 1 using fractional order $\alpha = [0.1, 0.2,\ldots , 1.0]$, (**a**) line loss minimization, $P_{Loss}$ (**b**) voltage deviation, $V_D$ and (**c**) overall cost, $C_{overall}$.

As a first case, the IEEE 30 bus system is adopted with the numerical values documented in Table 4. The initial operating cost without reactive power planning is $3.737016 \times 10^6$USD and the active power loss is 7.11 MW. Weak buses are identified through the power flow analysis method and according to that criterion the TCSC are installed in the 5th, 28th, 25th, and 41th lines. Using the VCPI method, the SVC are installed at the 4th, 20th, 22th and 28th buses. After that, the FO-DPSO is implemented for computing the optimal settings of the SVC, TCSC, tap changer position and reactive power generation, while considering all the fitness functions. The number of particles is taken as 80. The learning behavior of the FO-DPSO for line loss $P_{Loss}$ minimization using the orders $\alpha = [0.1, 0.2, \ldots , 1.0]$ is depicted in Fig. 4a, where the minimum loss is obtained at $\alpha$ = 0.6. The learning behavior for the voltage deviation and overall cost function are shown in Fig. 4b,c, respectively. The optimized settings of control variables including reactive power generation, tap value, size of the TCSC and SVC by mean of the FO-DPSO, are listed in Table 5 along with different optimization methods. The results of the FO-DPSO can be compared with those exhibited by other schemes including the GWO^30^, WOA^30^, QOGWO^30^, DE^23^, fuzzy DE^23^, SPSO^31^, APSO^31^, EPSO^31^ and QODE^30^ algorithms. The corresponding results are documented in Table 5, where one can observe that the FO-DPSO evaluated for the minimum losses, and compares well with the other optimization schemes.

**Table 4:** Description of the test case 1.

Index	Data	Quantity	Description
1	Generating units	6	At buses 1, 2, 5, 8, 11 and 13
2	Transformers	4	At branch 4–12, 6–10, 6–9 and 28–27
3	Transmission lines	41	–
4	Slack/reference bus	1	Bus number 1
5	Base MVA	–	100
6	$Q_{D}$	–	1.262 MVAR
7	$P_{D}$	–	2.834 MW
8	Shunt capacitors	2	At buses 10th and 24th

**Table 5:** Results generated by the FO-DPSO and other schemes for ORPD with FACTS during case 1.

Control	Reported									Proposed
Variable	SPSO^31^	APSO^31^	EPSO^31^	Fuzzy-DE^23^	GWO^30^	DE^23^	QODE^30^	QOGWO^30^	WOA^30^	FO-DPSO
$Q_G$ (2)	0.6	0.0	0.6	0.0	− 0.0108	− 0.5090	0.3535	0.0339	0.6	0.7494
$Q_G$ (5)	0.0	0.0	0.0	0.6	− 0.1151	− 0.1307	0.2365	− 0.0027	0.6250	0.4392
$Q_G$ (8)	0.0	0.0	0.0	0.25	0.1228	0.3955	0.4462	0.2907	0.5	0.3879
$Q_G$ (11)	0.4	0.4	0.4	0.0416	0.0797	0.3031	0.3497	0.0515	0.0029	0.2656
$Q_G$ (13)	0.0	0.0	0.0	0.0	0.2117	0.0474	0.2460	0.2342	0.0177	0.8470
T (11)	0.9	0.9	0.9439	0.9787	0.9	0.9021	0.9012	0.9	0.9	0.9889
T (12)	0.9	0.9501	0.9	0.9	0.9295	0.9658	0.9514	0.9452	0.9448	1.0072
T (15)	0.9	0.9180	0.9	0.9370	0.9	0.9007	0.9004	0.9	0.9	0.9974
T (36)	0.9223	0.9330	0.9326	0.9157	0.9289	0.9211	0.9278	0.9271	0.9285	0.9793
TCSC (1)	0.1463 (25)	0.1463 (25)	0.1463 (25)	0.0	0.08 (25)	0.08 (25)	0.0618 (25)	0.08 (25)	0.08 (25)	0.1113
TCSC (2)	0.0419 (41)	0.0419 (41)	0.0419 (41)	0.0	0.08 (41)	0.08 (41)	0.1588 (41)	0.08 (41)	0.08 (41)	0.2734
TCSC (3)	0.1049 (28)	0.1049 (28)	0.1049 (28)	0.0	0.08 (28)	0.0797 (28)	0.1999 (28)	0.08 (28)	0.08 (28)	0.0901
TCSC (4)	0.1388 (5)	0.1388 (5)	0.1368 (5)	0.0	0.08 (5)	0.08 (5)	0.0116 (5)	0.08 (5)	0.08 (5)	0.1348
SVC (1)	0.0 (7)	0.0 (7)	0.0 (7)	0.0	0.0368 (22)	0.0663 (22)	0.0797 (22)	0.0589 (22)	0.0524 (22)	0.1999
SVC (2)	0.0 (15)	0.0 (15)	0.0 (15)	0.0	0.1707 (04)	0.1492 (04)	0.08 (04)	0.12 (04)	0.1544 (04)	0.2
SVC (3)	0.0 (17)	0.0 (17)	0.0 (17)	0.0	0.20 (28)	0.1498 (28)	0.0789 (28)	0.20 (28)	0.2 (28)	0.2
SVC (4)	0.0840 (21)	0.0768 (21)	0.0 (21)	–	0.0049 (20)	0.0225 (20)	0.08 (20)	0.0091 (20)	0.0145 (20)	0.2
$P_{ Loss}$	0.05198	0.05092	0.05049	0.04745	0.04929	0.04881	0.0528	0.06331	0.06333	0.04683
$C_{overall}$	2.7324E+06	2.6767E+06	2.6541E+06	2.494E+06	2.5910E+06	2.5658E+06	2.7755E+06	3.3279E+06	3.3289E+06	2.4528E+08

The FO-DPSO computes real power loss of 0.04683 p.u and an operating cost as $2.4528 \times 10^6$ USD, that is inferior values with 0.0243 p.u and $1.284216 \times 10^6$ USD less than the base case, respectively. The comparative analysis for the line loss and operating cost reduction in relation with the other cases can be seen in Table 6. We observe that the strategy for ORPD incorporating FACTS provides a better solution than the other optimization mechanisms in terms of minimum losses and minimum overall operating cost.

To highlight the optimization strength of presented technique, we have also applied fractional evolutionary computing FO-DPSO to solve the ORPD problem in IEEE 30 bus system with 13 control variables without FACTS devices. The results are documented in Table 7. We verify that the results yielded by FO-DPSO are superior to the state of art solvers reported in literature including MICA-IWA^56^, PSO^57^, MFO^56^, HSA^57^, ICA^56^, GA^58^, IWO^59^, DE^57^, GWO^60^ and NMFLA^61^. The line loss reduction from presented scheme as compared to counterparts can be seen in Table 8 where it is endorsed that FO-DPSO is the best.

**Table 6:** Comparative results of loss and overall cost reductions.

Methods	$P_{Loss}$ (p.u)	$C_{overall}$ (USD)	Loss reduction (p.u)	Cost reduction (USD)
Base case	0.0711	3737016	–	–
Fuzzy-DE^23^	0.04745	2.49E+06	0.0236	1.24E+06
SPSO^31^	0.05198	2.73E+06	0.0191	1.00E+06
PSO^31^	0.05092	2.68E+06	0.0202	1.06E+06
EPSO^31^	0.05049	2.65E+06	0.0206	1.08E+06
DE^23^	0.04881	2.57E+06	0.0223	1.17E+06
QODE^30^	0.0528	2.78E+06	0.0183	9.62E+05
GWO^30^	0.04929	2.59E+06	0.0218	1.15E+06
QOGWO^30^	0.06331	3.33E+06	0.0078	4.09E+05
WOA^30^	0.06333	3.33E+06	0.0078	4.08E+05
Proposed	0.04683	2.45E+08	0.0243	1.28E+06

**Table 7:** Comparative study for the 30 bus network with 13 decision variables without FACTS.

Control Variables	MICA-IWO	PSO	MFO	HSA	ICA	GA	IWO	DE	GWO	NMFLA	FO-DPSO
V1	1.07972	1.0313	1.1	1.0726	1.0785	1.0721	1.06965	1.095319	1.1	1.1000	1.01
V2	1.07055	1.0114	1.0946	1.0625	1.06943	1.063	1.06038	1.085946	1.096149	1.0945	1.04231
V5	1.04836	1.0221	1.0756	1.0399	1.06943	1.0377	1.03692	1.062628	1.080036	1.0753	1.0401
V8	1.04865	1.0031	1.772	1.0422	1.04714	1.0445	1.03864	1.065076	1.080444	1.0773	1.0956
V11	1.07518	0.9744	1.0868	1.0318	1.03485	1.0132	1.02973	1.0266	1.093452	1.1000	1.0110
V13	1.07072	0.9987	1.1	1.0681	1.07106	1.0898	1.05574	1.014253	1.1	1.1000	1.0491
T6-9	1.03	0.97	1.04110	1.01	1.08	1.0221	1.05	1.017796	1.04	1.06	1.0610
T6-10	0.99	1.02	0.95007	1	0.95	0.9917	0.96	0.979277	0.95	0.92	0.9295
T4-12	1	1.01	0.95541	0.99	1	0.9964	0.97	0.977843	0.95	0.95	0.9665
T27-28	0.98	0.99	0.95754	0.97	0.97	0.971	0.97	1.008938	0.95	0.96	0.9555
Qc3	− 7	17	7.1032	34	− 6	5.3502	8	20.22359	12	0.08	8.4272
Qc10	23	13	30.796	12	36	36	35	9.584327	30	0.26	25.1542
Qc24	12	23	9.8981	10	11	12.4175	11	13.02992	8	0.10	9.2331
$P_{Loss}$(MW)	4.846	5.8815	4.608	5.109	4.849	4.8775	4.92	4.888081	4.613	4.6118	4.606

**Table 8:** $P_{Loss}$ reduction for the 30-bus model with MFO, MICA-IWO, GA, HSA, GWO, DE, NMSFLA having 13 decision/control variables without FACTS.

Items	Initial	MICA-IWO	GA	HSA	MFO	GWO	DE	NMSFLA	FO-DPSO
$P_{Loss}$(MW)	5.663	4.846	4.8775	5.109	4.608	4.613	4.888080765	4.6118	4.606
Loss reduction (%)	–	14.44	13.87	9.78	18.64	18.54	13.68	18.56	18.66

### Test case 2

**Figure 5:**
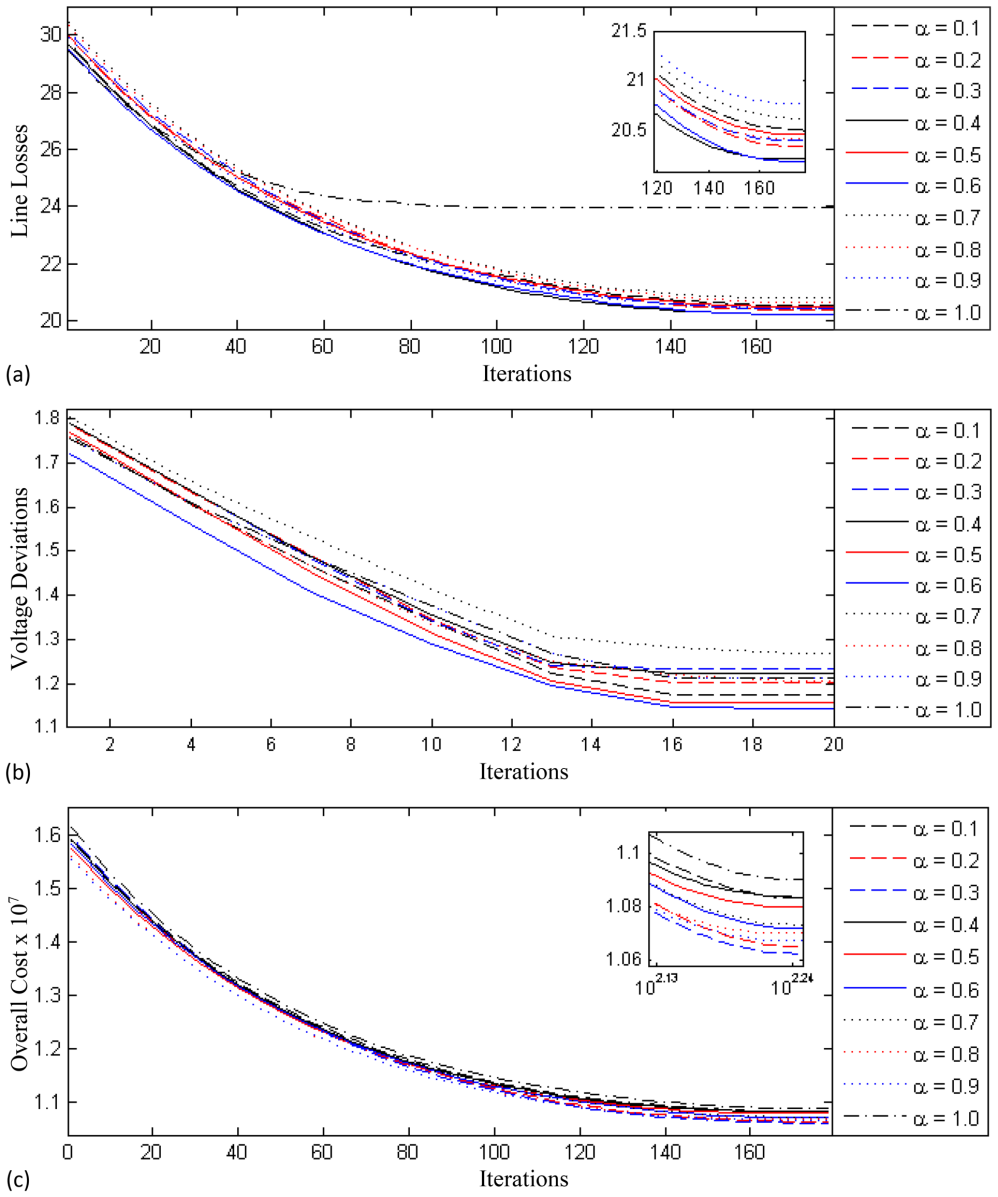
Learning curves for test case 2 using fractional order $\alpha = [0.1, 0.2,\ldots , 1.0]$, (**a**) line loss minimization, $P_{Loss}$, (**b**) voltage deviation, $V_D$ and (**c**) overall cost, $C_{overall}$.

The second test case addresses the $P_{Loss}$ of the IEEE 57 bus system. This standard power system comprises 80 transmission lines with the tap changing transformers installed at seventeen links, 7 generating stations synchronized at buses 1, 2, 3, 6, 8, 9 and 12, and three shunt capacitors. Bus# 1 represents the slack bus, that is the reference bus. The cumulative power demand for the real and reactive load is 12.5170 MW and 3.3570 MVAR, respectively, at base power of 100 MVA. Initially, the cost of operation is $1.471 \times 10^7$ USD and the $P_{Loss}$ is 27.99 MW without ORPD. The SVC are installed at the $23^{rd}$, 38th, 39th, and 48th, buses, that are identified as weak buses by means of the VCPI method. The TCSC are installed in 13th, 37th, 57th, and $61^{st}$ lines that are found as the weak links based on the power flow technique. The FO-DPSO is tested to evaluate the best values of the dependent variables, namely the size of the SVC and TCSC along with the tap changer position and reactive power generation while reducing the network evaluation fitness functions including $P_{Loss}$, $V_{D}$ and $C_{overall}$ minimization. The behavior of FO-DPSO using different differential orders $\alpha = [0.1, 0.2,\ldots , 1.0]$ can be seen in Fig. 5a for the line loss $P_{Loss}$, in Fig. 5b for the voltage deviation $V_D$, and in Fig. 5c for the overall cost $C_{overall}$. The superiority of the new approach is again endorsed when comparing the results of the FO-DPSO and those of the SPO^31^, APSO^31^, EPSO^31^, DE^23^, QODE^30^ and GWO^30^ algorithms. The results are listed in Table 9. The system operating cost and the real power loss reduction for different solvers is documented in Table 10 that highlights the significance of the FO-DPSO as both quantities are considerably smaller than those obtained when adopting other optimization strategies. Additionally, from these results, we verify that the $P_{Loss}$, $V_{D}$ and $C_{overall}$ converge smoothly and with a less iterations for the FO-DPSO with respect to the other optimization algorithms.

**Table 9:** Comparison of the FO-DPSO versus other schemes for ORPD with FACTS during case 2.

Control variables	Reported						Proposed
	QODE^30^	DE^23^	EPSO^31^	APSO^31^	GWO^30^	SPSO^31^	FO-DPSO
Qg(2)	− 0.0011	0.1504	0.5	0.1213	− 0.1258	0.5	0.6
Qg(3)	0.0348	0.2594	0.6	0.5754	0.1785	0.6	0.25
Qg(6)	0.1377	0.2239	0.25	0.25	0.1926	0.25	− 0.09
Qg(8)	0.1816	1.8451	0.2	0.2	− 0.103	0.2	0.5713
Qg(9)	0.0364	− 0.0129	0.09	0.09	0.0049	0.09	0.0529
Qg(12)	1.2359	1.3528	0	0	0.0026	0	1.475
T(19)	0.903	0.92	1.1	0.9	0.9145	0.9	0.903
T(20)	0.9135	0.9109	0.9	0.9152	0.9041	0.9	0.9
T(31)	1.0203	1.008	1.1	1.0892	1.0385	1.0128	1.05
T(35)	1.0203	1.008	1.1	0.9	1.0385	0.9	0.912
T(36)	0.9531	0.9391	0.9	0.9474	0.9263	0.9	0.9
T(37)	1.009	1.0498	1.0109	1.0281	1.0336	1.0203	1.05
T(41)	0.9037	0.9019	0.9	0.9021	0.9	0.9	0.9
T(46)	0.9871	0.9152	1.1	0.9	0.9058	0.9	0.9034
T(54)	0.9481	0.9281	0.9	0.9558	0.9109	0.9	0.9001
T(58)	0.9008	0.9003	0.9	0.9	0.9002	0.9	0.9
T(59)	1.0496	1.0483	0.9	0.9	1.05	0.9	1.05
T(65)	0.9004	0.9095	0.9	0.9456	0.9	0.9	0.9
T(66)	0.9123	0.9156	1.1	0.9274	0.9051	0.9	0.911
T(71)	0.9123	0.9156	1.1	0.9274	0.9051	0.9	0.9
T(73)	1.0394	1.0446	1.1	1.1	1.0371	1.1	1.005
T(76)	1.0488	0.9673	0.9	1.0357	0.9905	0.9	0.9
T(80)	0.9245	0.9044	0.9	0.9	0.9024	0.9	0.913
TCSC(1)-37	0.0941	0.0888	0.0331	0.0331	0.0242	0.0331	0.11
TCSC(2)-13	0.94	0.071	0.0334	0.0304	0.0115	0.0304	0.071
TCSC(3)-61	0.11	0.1098	0.0163	0.0163	0.11	0.0163	0.107
TCSC(4)-57	0.109	0.1073	0.041	0.041	0.11	0.041	0.11
SVC(1)-23	0.1794	0.1982	0	0	0.2	0	0.2
SVC(2)-48	0.1995	0.1939	0	0	0.2	0	0.2
SVC(3)-38	0.1954	0.1981	0.4397	0.5099	0.1999	0.3945	0.2
SVC(4)-39	0.193	0.1792	–	–	0.1986	–	0.2
$P_{loss}$	0.2097	0.2097	0.2275	0.2231	0.2097	0.221	0.206
$C_{overall}$	1.1024E+7	1.1021E+7	1.203E+7	1.179E+7	1.102E+7	1.168E+7	1.08E+07

**Table 10:** Comparative results of the loss and overall cost reduction.

Methods incorporating FACTS	$P_{Loss}$ (p.u)	$C_{overall}$(USD)	Loss Reduction (p.u) (A1 - A)	Cost reduction, USD (B1 - B)
Base case	0.27E−01 (A1)	1.47E+07 (B1)	–	–
SPSO^31^	2.21E−01	1.17E+07	5.89E−02	3.03E+06
EPSO^31^	2.28E−01	2.28E−01	1.20E+07	5.24E−02
APSO^31^	2.23E−01	2.23E−01	1.18E+07	5.68E−02
DE^23^	2.10E−01	2.10E−01	1.10E+07	7.02E−02
GWO^30^	2.10E−01	2.10E−01	1.10E+07	7.02E−02
QOGWO^30^	2.07E−01	2.07E−01	1.09E+07	7.27E−02
QODE^30^	2.10E−01	2.10E−01	1.10E+07	7.02E−02
FO-DPSO	2.06E−01	2.06E−01	1.08E+07	7.39E−02

### Test case 3

**Figure 6:**
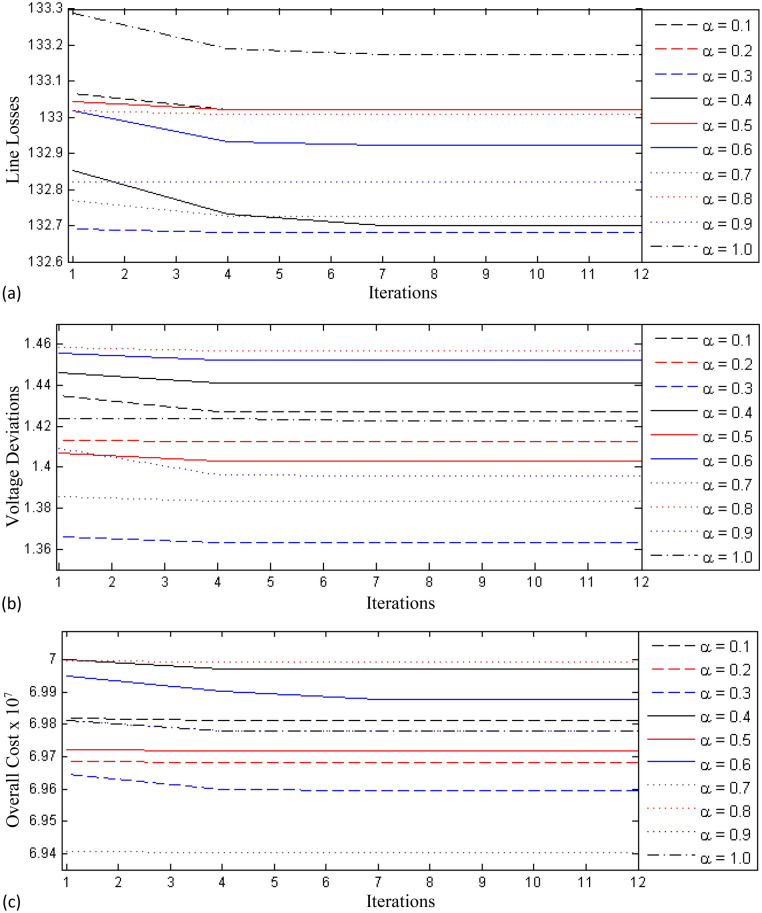
Learning curves for test case 3 (IEEE 118 bus system) using fractional order $\alpha = [0.1, 0.2,\ldots , 1.0]$ (**a**) line loss minimization, $P_{Loss}$, (**b**) voltage deviation, $V_D$ and (**c**) overall cost, $C_{overall}$.

The 3rd test case consists of the standard IEEE 118 bus network for validating the FO-DPSO in the case of large scale power systems. This system contains 186 lines, 9 transformer, 64 load buses, and 54 generator buses. Here, the system restrictions and settings were derived from^57^. Bus# 1 is considered as the slack/reference bus and the base power is 100 MVA. The FO-DPSO is tested to evaluate the optimum values of the dependent variables while reducing the fitness functions. The effectiveness of FO-DPSO is again endorsed by mean of a comparative analysis between the results of the proposed technique and those provided by the OGSA, WCA and NGBWCA^56,57^. The results are documented in Table 11, one may observe that FO-DPSO achieved losses inferior than those provided by other optimization mechanisms. The setting of the control variables for the FO-DPSO and other optimization strategies can also be seen in Table 11. The comparative learning behavior of the FO-DPSO using different differential orders $\alpha = [0.1, 0.2,\ldots , 1.0]$ can be seen in Fig. 6a for line loss (i.e $P_{loss}$), in Fig. 6b for voltage deviation (i.e., $V_{D}$) and in Fig. 6c for overall cost (i.e., $C_{overall}$). We can verify again that, all the minimization functions converge for a smaller number of iterations and evolve more smoothly for the FO-DPSO with respect to other methods.

It is important to mention that the statistical analysis has been performed for 100 independent trials and for each independent run the population (i.e., the swarm), is initialized with pseudo random real numbers between the allowable bounds of decision variables. Different initial populations/swarms are used for each independent trial and the robustness of FO-DPSO is endorsed by the optimization of the control variable with reasonable accuracy for each trail. The difference in performances depicted by Figs. 4, 5 and 6, is due to some better initial population which is completely formulated on random process. Therefore, the main concern/intention for multiple runs is to prove/certify the reliability, effectiveness and stability of the FO-DPSO on standard ORPD problems.

**Table 11:** Comparison of control variables for test case 3 with OGSA, WCA, NGBWCA from proposed technique.

Variable	OGSA	WCA	NGBWCA	FO-DPSO	Variable	OGSA	WCA	NGBWCA	FO-DPSO
Generator voltage									
V1	1.0388	1	1.0002	0.9655	V100	1.0032	1.0029	1.0021	1.0846
V4	0.9872	1.0194	1.0202	1.0378	V103	0.9843	1.0502	0.9998	0.9677
V6	0.9925	0.9996	0.9936	1.1	V104	0.988	0.9872	0.9852	1.0735
V8	0.9905	0.9812	0.9771	1.069	V105	1.0003	0.9992	0.9994	1.079
V10	0.9919	1.0031	1.0051	1.0426	V105	1.0033	1.0136	1.0198	1.0974
V12	1.0077	1.0131	1.012	1.0758	V110	1.004	1.0043	1.0152	1.1
V15	1.0034	0.9859	0.9853	1.0209	V111	1.0331	1.0247	1.0241	1.058
V18	0.9773	1.0575	1.0557	1.0103	V112	0.9877	1.0023	1.0023	0.9995
V19	1.0324	1.0203	1.019	1.0595	V113	0.9705	0.9825	0.9951	1.0211
V24	1.0285	1.0201	1.0197	1.0548	V116	1.027	0.9976	0.997	1.0829
V25	0.9705	1.0246	1.0108	0.9978	Transformer tap ratio				
V26	1.0175	0.9883	0.9954	1.0031	T8	0.9841	0.9956	1.0484	1.0792
V27	1.0117	1.0164	1.0204	1.0695	T32	1.0377	0.9712	0.9511	0.9973
V31	1.0014	0.9976	0.999	1.0919	T36	0.9573	1.034	1.0312	1.075
V32	0.9988	0.9913	0.9877	1.0965	T51	0.9952	0.9817	0.9811	0.9954
V34	1.0158	1.0027	1.0211	1.0962	T93	0.9622	1.0212	1.0224	0.9888
V36	0.9916	0.9687	0.9656	0.9819	T95	1.032	0.9976	0.9972	1.0071
V40	1.0132	1.0002	1.0031	1.0294	T102	1.0137	1.0021	1.0249	1.005
V42	0.9892	1.0115	1.0012	1.0616	T107	0.9795	0.9679	0.9621	0.9904
V46	1.0607	1.0531	1.0512	1.0384	T127	0.9985	1.0212	1.0102	1.0309
V49	1.0031	1.0026	1.0001	1.0154	Capacitor banks				
V54	1.0236	1.0231	1.0227	1.0357	QC-5	− 0.2403	− 0.1427	− 0.1413	0.9772
V55	1.0176	1.0346	1.0323	0.9959	QC-34	0.0371	0.0215	0.0212	0.9658
V56	1.0149	1.0131	1.0139	1.0802	QC-37	− 0.0437	− 0.1390	− 0.1319	0.9804
V59	1.0584	1.0099	1.0084	1.0275	QC-44	0.0375	0.0712	0.0781	18.1179
V61	0.9829	1	1.0001	0.982	QC-45	0.04	0.0452	0.0459	− 24.2592
V62	1.0562	1	1.0027	1.0944	QC-46	0.0749	0.0549	0.0711	− 7.7363
V65	0.9724	0.9694	0.9681	1.0755	QC-48	0.0796	0.1076	0.1002	22.8106
V66	1.002	1.0175	1.0143	1.0852	QC-74	0.0883	0.0084	0.0082	− 4.3584
V69	0.9827	1.0158	0.9995	1.091	QC-79	0.1218	0.0197	0.019	− 9.5234
V70	0.9997	0.9814	0.9721	1.0043	QC-82	0.038	0.1435	0.1417	28.0814
V72	1.0123	0.991	0.9987	1.0104	QC-83	0.0627	0.0813	0.0921	1.4103
V73	0.996	1.0313	0.9946	1.0785	QC-105	0.083	0.1146	0.116	6.8655
V74	1.0232	1.0002	1.0212	1.0794	QC-107	0.0459	0.0279	0.0242	− 27.6663
V76	1.0015	1.0097	1.0024	1.05	QC-110	0.0221	0.0276	0.0257	− 27.6663
V77	1.0124	1.03	1.0122	1.0357					
V80	1.0226	1.0124	0.9998	1.0905					
V85	1.0117	1.0112	1.0205	1.1					
V87	1.0058	0.9997	1.0002	1.0159					
V89	1.0076	1.0087	1.0002	1.0497					
V90	0.9753	1.0145	1.0182	0.9941					
V91	0.9836	0.9934	0.9879	1.0182		OGSA	WCA	NGBWCA	F0-DPSO
V92	1.0272	0.9994	0.9999	0.9747					
V99	0.9612	1.0712	1.0672	1.0647	$P_{loss}$, MW	157.72	165.71	152.31	132.5

## Statistical analysis

**Figure 7:**
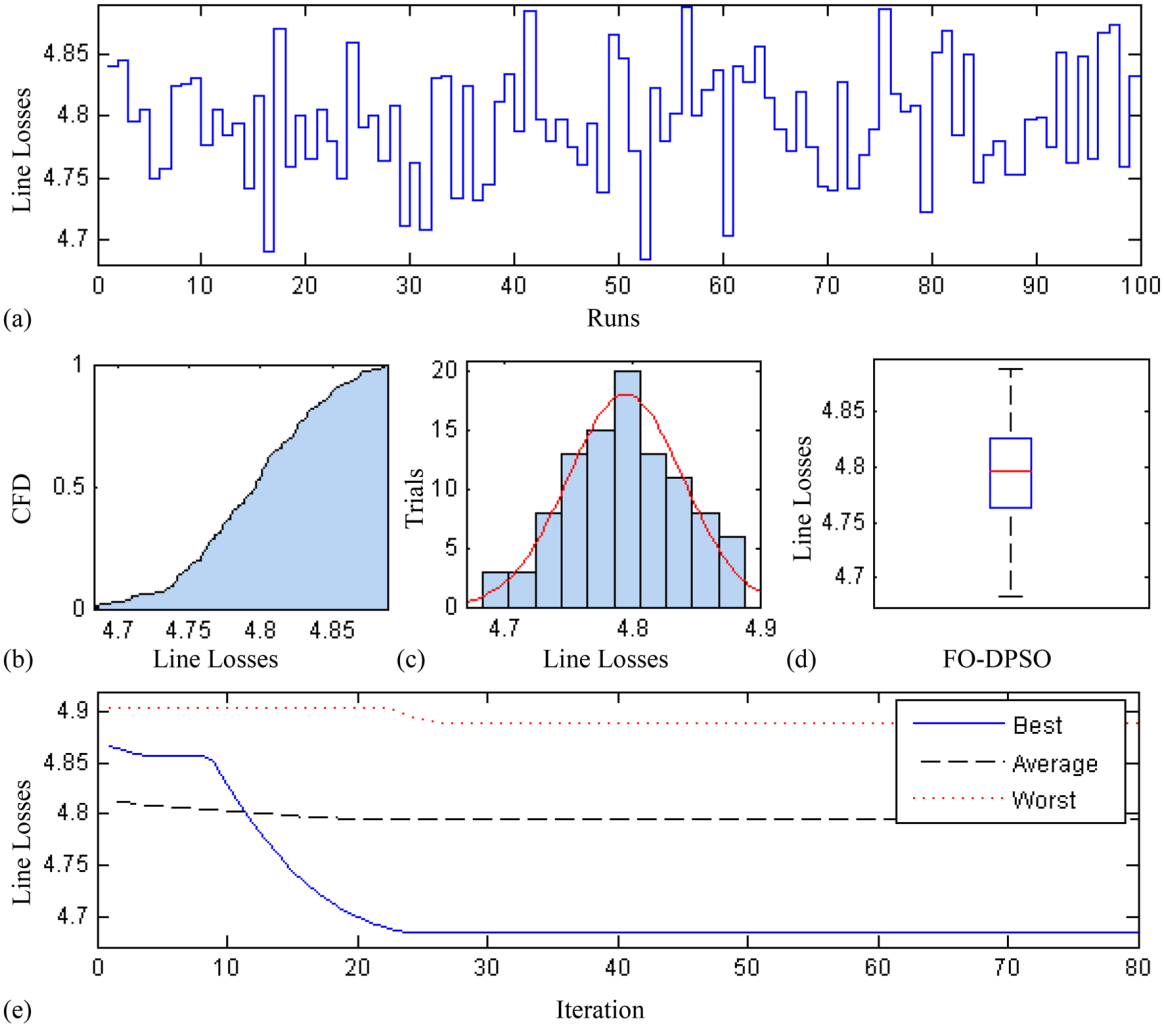
Statistical analysis for test case 1: power loss minimization during 100 free runs, (**a**) minimum fitness comparison, (**b**) CDF, (**c**) histogram analysis, (**d**) fitness boxplot, (**e**) learning behavior.

**Figure 8:**
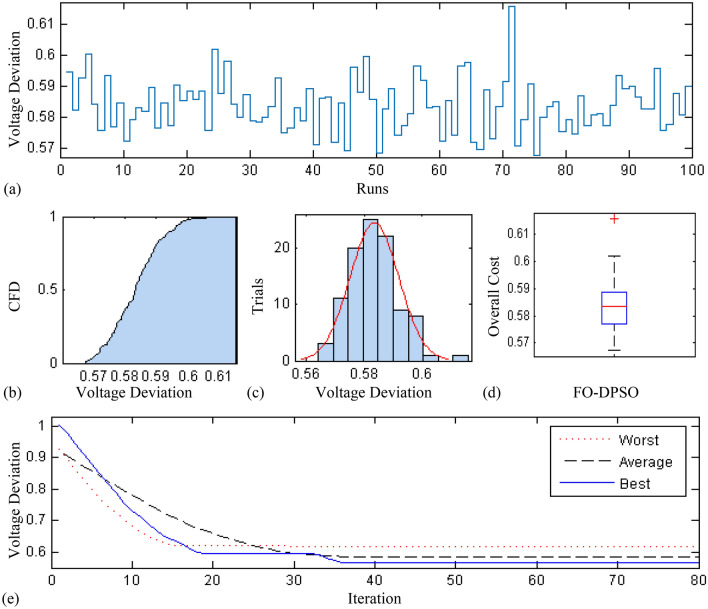
Statistical Analysis for test case 1: voltage deviation during 100 free runs, (**a**) minimum fitness comparison, (**b**) CDF, (**c**) histogram analysis, (**d**) fitness boxplot, (**e**) learning behavior.

**Figure 9:**
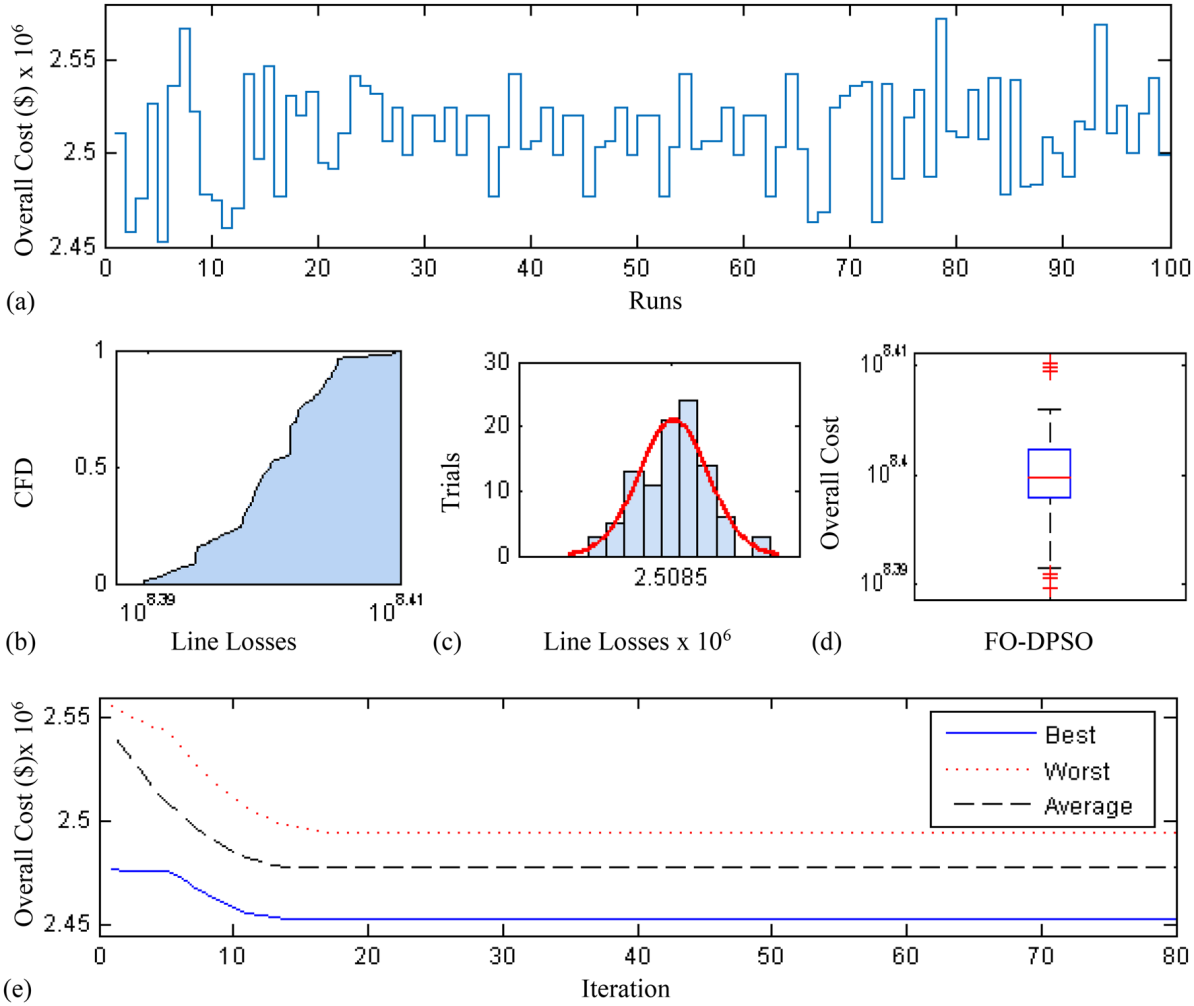
Statistical analysis for test case 1: overall cost minimization during 100 free runs, (**a**) minimum fitness comparison, (**b**) CDF, (**c**) histogram analysis, (**d**) fitness boxplot, (**e**) learning behavior.

**Figure 10:**
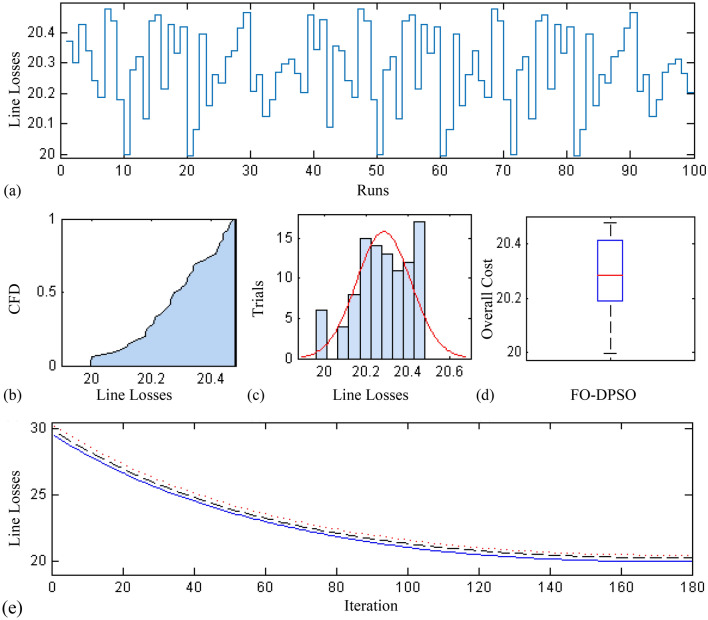
Statistical analysis for test case 2: fitness in Section power loss minimization during 100 free runs, (**a**) minimum fitness comparison, (**b**) CDF, (**c**) histogram analysis, (**d**) fitness boxplot, (**e**) learning behavior.

**Figure 11:**
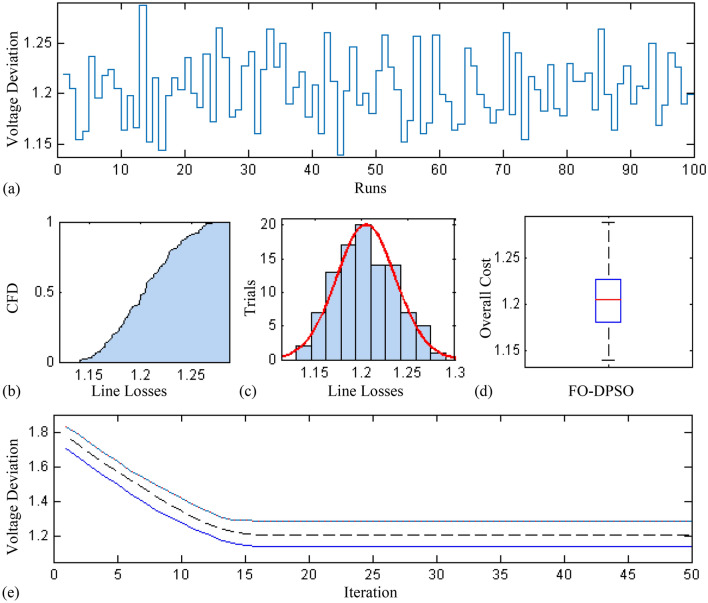
Statistical analysis for test case 2: voltage deviation during 100 free runs, (**a**) minimum fitness comparison, (**b**) CDF, (**c**) histogram analysis, (**d**) fitness boxplot, (**e**) learning behavior.

**Figure 12:**
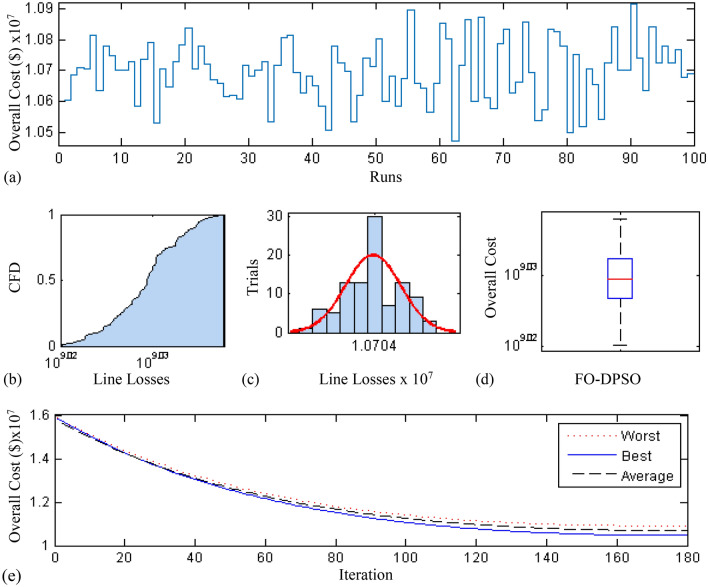
Statistical analysis for test case 2: overall cost minimization during 100 free runs, (**a**) minimum fitness comparison, (**b**) CDF, (**c**) histogram analysis, (**d**) fitness boxplot, (**e**) learning behavior.

**Figure 13:**
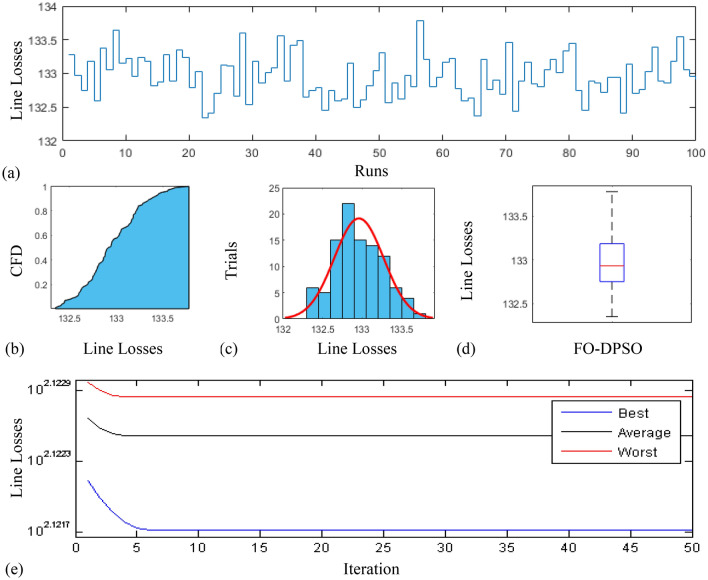
Statistical analysis for test case 3: power loss minimization during 100 free runs, (**a**) minimum fitness comparison, (**b**) CDF, (**c**) histogram analysis, (**d**) fitness boxplot, (**e**) learning behavior.

**Figure 14:**
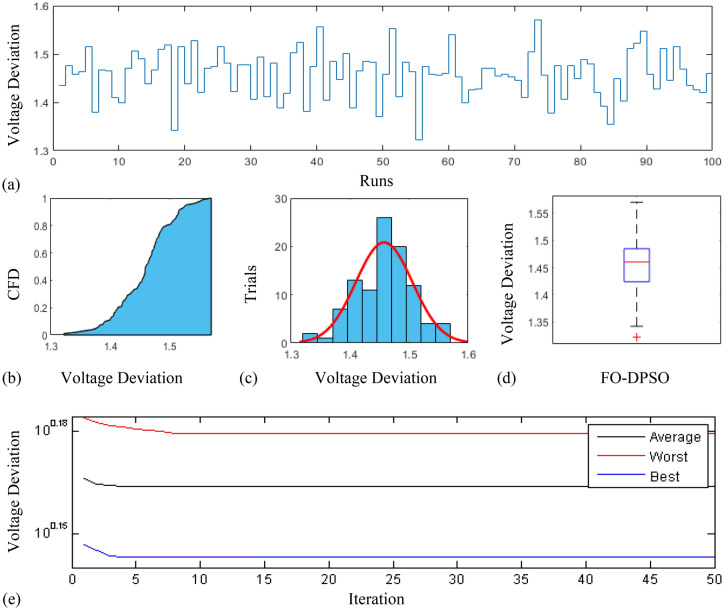
Statistical analysis for test case 3: voltage deviation during 100 free runs, (**a**) minimum fitness comparison, (**b**) CDF, (**c**) histogram analysis, (**d**) fitness boxplot, (**e**) learning behavior.

**Figure 15:**
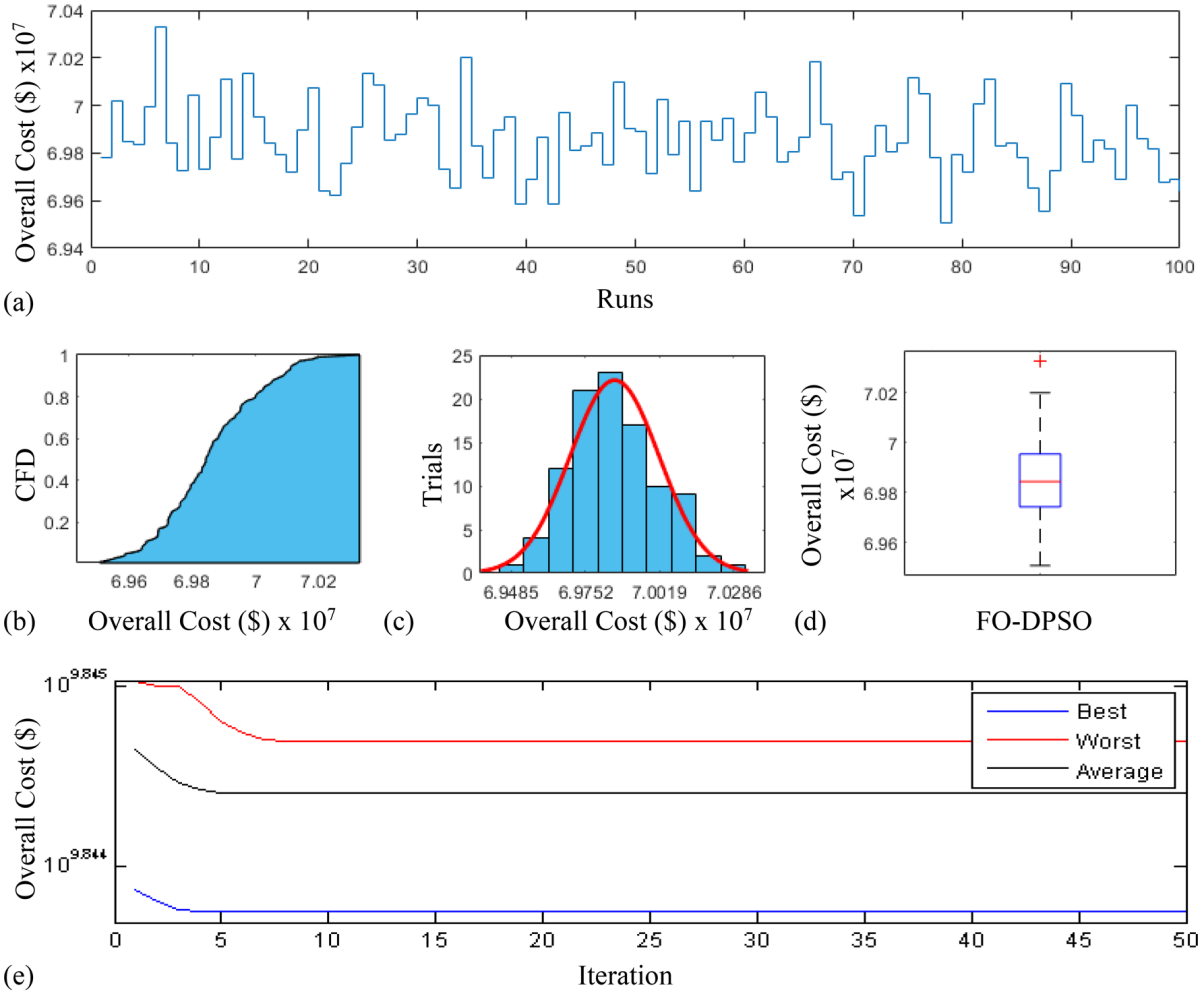
Statistical analysis for test case 3: overall cost minimization during 100 free runs, (**a**) minimum fitness comparison, (**b**) CDF, (**c**) histogram analysis, (**d**) fitness boxplot, (**e**) learning behavior.

In this section, a comprehensive statistical investigation is conducted to demonstrate consistent implications of the FO-DPSO evolution for all the test cases and the three fitness functions of ORPD problems. The performance of the FO-DPSO with fractional order $\alpha$ = 0.6 revealed the best results on average among the set $\alpha = [0.1, 0.2,\ldots , 1.0]$ for the IEEE 30 and IEEE 57 bus systems, while fractional order $\alpha$ = 0.3 for the IEEE 118 bus system. Therefore, a sample of 100 independent runs are conducted with the FO-DPSO using $\alpha$ = 0.6 for the test cases 1 and 2, while $\alpha$ = 0.3 for the test case 3, considering $P_{Loss}$, $V_{D}$ and $C_{overall}$ minimization functions as the objectives of ORPD system incorporating FACTS. The statistics from the three fitness measures, in term of stair plot, probability metric for box plot, cumulative distribution function (CDF), histogram, and convergence curves for the best, worst and mean gauges, are demonstrated in Figs. 7, 8 and 9, Figs. 10, 11, 12 and Figs.13, 14 and 15 for the IEEE 30, 57 and 118 bus networks, respectively. The minimum value of the fitnesses in all the test cases are depicted in Figs. 7a, 8a, 9a, 10a, 11a, 12a, 13a, 14a and 15a. The probability plots for the CDF are illustrated in Figs. 7b, 10b and 13b showing that 80% of the independent flights yield line losses inferior to 4.85 MW, 20.4 MW and 132.7 MW for the test cases 1, 2 and 3, respectively. The histograms represented in Figs. 7c, 8c, 9c, 10c, 11c, 12c, 13c, 14c and 15c reveal that most of the autonomous simulations of the FO-DPSO yield minimum values of the three fitness functions. The values of box plots in Figs.  7d, 10d and 13d demonstrate that the median of line losses is approximately 4.8 MW and 20.3 MW for the three test cases, respectively. The learning behavior for the best, average and worst cases are included in Figs. 7e, 8e, 9e, 10e, 11e, 12e, 13e, 14e and 15e that demonstrate the consistency of the FO-DPSO for an effective optimization. In brief, all statistics of the ORPD cases demonstrate the stability, robustness, and consistency of the FO-DPSO as a significant, reliable and accurate optimization strategy.

**Figure 16:**
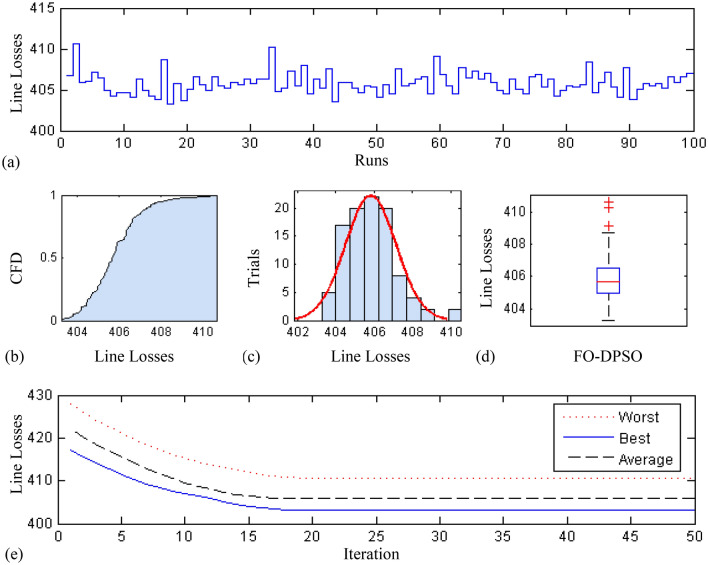
Statistical analysis for 300 bus system: power loss minimization during 100 free runs, (**a**) minimum fitness comparison, (**b**) CDF, (**c**) histogram analysis, (**d**) fitness boxplot, (**e**) learning behavior.

The scalability of the FO-DPSO is further extended on a very large power system i.e. IEEE 300 bus which contains 304 transmission lines, 60 tap changing transformers, and 69 generators. This study proposes the earliest solution for a single objective of ORPD which is line loss minimization, $P_{Loss}$. As IEEE-300 bus system is challenging problem, the solutions for reactive power dispatch problems published in the literature are very rare, and so the comparison of results is not possible at this stage. However, when the FO-DPSO is applied to tune the variables, better and consistent results than the base case values are obtained. The computed $P_{Loss}$ from proposed strategy are 403.259 MW, which are 1.2% less than the base case i.e. 408.316MW. The statistical results obtained for 100 independent trial are depicted in Fig. 16. The Fig. 16a demonstrates that for 95 times, the minimum fitness values obtained by the FO-DPSO are below the base case value (408.316 MW). The CDF based probability plot in Fig. 16b reveals that 90% of the independent runs computed $P_{Loss}$ values less than 407 MW. The histogram represented in Fig. 16c shows that maximum of the independent trials provide minimum gauge of the fitness function. The values of box plot in Fig. 16d reveal that median of $P_{Loss}$ is approximately 405.8 MW with relative small spread of data. The learning curves for the best, average and worst cases can be seen in Fig. 16e that demonstrate the consistency of the FO-DPSO for an effective computation.

**Figure 17:**
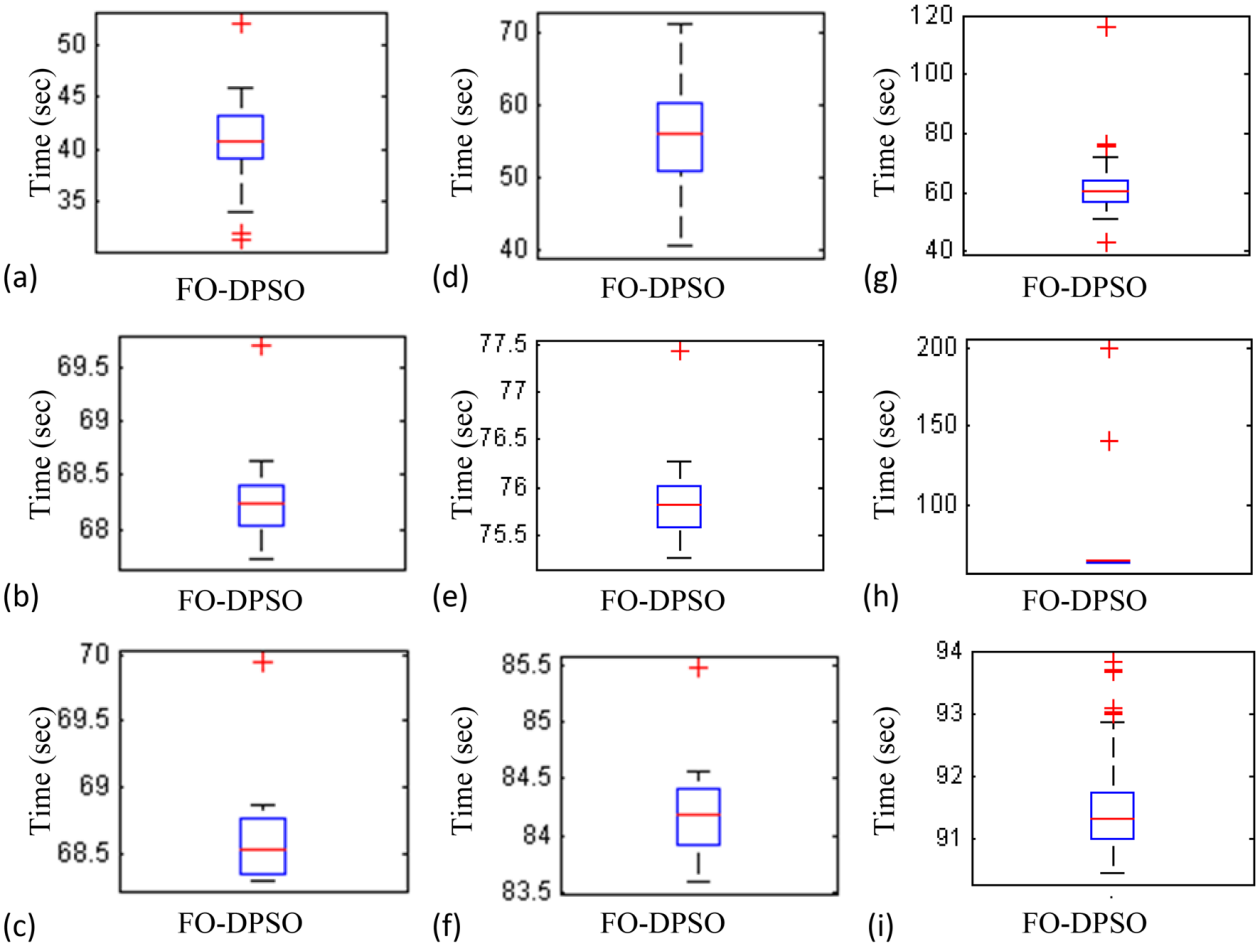
Box plot (**a**) case1; line losses, (**b**) case1; voltage deviation, (**c**) case1; overall cost, (**d**) case2; line losses, (**e**) case2; voltage deviation, (**f**) case2; overall lost (**g**) case3; line losses, (**h**) case3; voltage deviation, (**i**) case3; overall cost.

The time complexity of FO-DPSO is presented in the box plots of Fig. 17 for all the evaluated fitnesses. The calculated time of the algorithm execution for 100 independent runs in term of median gauge adopting test case 1 for $P_{Loss}$, $V_{D}$, and $C_{overall}$ minimization are around 42 s, 68.3 s and 68.5 s, respectively, for case 2 the respective values are around 55 s, 75.75 s, 84.25 s, while for case 3 the values are around 60 s, 80.3 s and 91.5 s, respectively. The small difference between the calculated time for each independent trial (i.e., results of the first quartile and third quartile) show the smooth and consistency operation of proposed FO-DPSO technique for solving the ORPD problems.

The computational complexity of the FO-DPSO is also compared with the other algorithms implemented to solve the ORPD problems. The reported results of time complexity of seeker optimization algorithm (SOA), simple GA (SGA)^62^, PSO, multi agent PSO (MAPSO)^63^, improved evolutionary programming (IEP), evolutionary programming EP^64^, self-adaptive real coded GAs, SARGA^65^ and the FO-DPSO are documented in Table 12 along with the available specification of the system and number of independent trials used for the analysis. The results show that there is no noticeable variation in the computational time requirements of the FO-DPSO versus those for the rest of the algorithms. However, the complexity performance is difficult to compare because reported results are based on machines with different hardware specification, i.e., RAM, CPU, cloud and parallel processing platform, operating algorithms (i.e., swarm intelligence, evolutionary computing) with different initial settings of swarm size, population, flights and generations, and software environment (i.e., operating systems, MATLAB, MATHEMATICA, etc.).

**Table 12:** Analysis of time complexity with state of art counter parts.

S. no.	Reference	Specifications	Bus system	Average time (s)
1	SOA	Matlab 7, Pentium 4,	IEEE-57,	391.32
	Run=30	CPU 2.4 GHz, 512 MB RAM	IEEE-118	–
2	SGA	Matlab 6.5, Pentium 4,	IEEE-57,	156.34
	Run=30	CPU N.A, RAM N.A	IEEE-118	335.54
3	PSO	Matlab 6.5, Pentium 4,	IEEE-57,	59.21
	Run=50	CPU N.A, RAM N.A	IEEE-118	144.46
4	MAPSO	Matlab 6.5, Pentium 4,	IEEE-57,	41.93
	Run=50	CPU N.A, RAM N.A	IEEE-118	119.35
5	IEP	Pentium 3 750	IEEE-118	77.35–142.8
6	EP	Matlab 6.5, Pentium 4,	IEEE-14,	72–78
		CPU N.A, 128 MB RAM	IEEE-30	103–118
7	SARGA	Matlab 6.5, Pentium 4,	IEEE-30,	54–66
		CPU N.A, 128 MB RAM	IEEE-118	87–101
8	Proposed	Matlab 2016, Core i 7,	IEEE-30,	42
	Run=100	CPU 3.4 GHz, 8 GB RAM	IEEE-57	55
			IEEE-118	60

## Conclusions

A fractional evolutionary computing algorithm was designed to solve the ORPD problem in power systems using shunt and series FACTS devices. The FO-DPSO was explored for the minimization of the active power losses and the operating costs, together with the installation cost of FACT, while the voltage profile is maintained within the allowable limits through minimizing voltage deviation index in the standard IEEE-30, 57 and 118 bus systems. The results using the FO-DPSO are compared with those reported in the literature adopting the GWO, WOA, QOGWO, DE, fuzzy DE, SPSO, APSO, EPSO, QODE, OGSA, WCA and NGBWCA schemes. The results demonstrated the superior performance of the FO-DPSO for all objectives of ORPD with FACTS devices. The validation of the FO-DPSO is supported by statistics that include the probability distribution functions, histogram and boxplot representations as measures of the central tendency and diversity indices for ORPD problems solved for the standard test systems.

In future, one may exploit the strength of the fractional swarming/evolutionary computing paradigm as an alternative optimization solver for multi-model nonlinear problems including robust wind power prediction^66^, forecasting of air temperature^67^, design of optical metasurfaces^68^, nonlinear active noise control^69^, parameter estimation of photovoltaic models^70^, optimization of design for desalination plant^71^, multi-objective classification problems^72^ and prediction of blast-induced ground vibrations^73^. In addition, the power system performance should be investigated further by incorporating the second generation FACTS devices including the STATCOM, UPFC and TCPS while operating in steady and dynamic states by exploiting the optimization legacy of proposed fractional swarming technique with orders $\alpha \le 0$, $0<\alpha <1$ and $\alpha > 1$. The selection of appropriate fractional order $\alpha$ in FO-DPSO with theoretical justification of the physics for a particular optimization problem looks promising to be explore by research community as a further related work.
